# New combinations in Neotropical Thelypteridaceae

**DOI:** 10.3897/phytokeys.57.5641

**Published:** 2015-12-02

**Authors:** Alexandre Salino, Thaís E. Almeida, Alan R. Smith

**Affiliations:** 1 Departamento de Botânica, Universidade Federal de Minas Gerais, Av. Antônio Carlos, 6627 – Belo Horizonte, Minas Gerais, Brazil. Caixa Postal 486, CEP 30123-970; 2 Programa de Ciências Naturais, Instituto de Ciências da Educação – Universidade Federal do Oeste do Pará, Avenida Marechal Rondon, s/n, Campus Rondon – Santarém, Pará, Brazil 68040-070; 3 University Herbarium, University of California, 1001 Valley Life Sciences Bldg. # 2465, Berkeley, CA 94720-2465, USA

**Keywords:** Amauropelta, ferns, Goniopteris, Steiropteris, Thelypteris

## Abstract

288 new combinations of Neotropical Thelypteridaceae taxa are proposed in order to recognize monophyletic genera, based on the results of the most recent molecular phylogeny of the family, as well as the morphological uniformity of characters for each genus. The new nomenclatural combinations correspond to 186 Amauropelta taxa, 77 species of Goniopteris, and 25 Steiropteris taxa. A key to all native Neotropical genera of the family is also presented.

## Introduction

The Thelypteridaceae, comprising approximately 1,000 species, is one of the largest families of ferns (Smith et al. 2006). It has a cosmopolitan distribution, with most species occurring in tropical and subtropical regions (Smith 1992, Smith et al. 2006, 2008). The family is monophyletic as usually construed (Smith and Cranfill 2002, Schuettpelz and Pryer 2007, He and Zhang 2013, and Almeida et al. 2015). However, the classification within the family is controversial, with authors recognizing different numbers of genera: 32 (Pichi Sermolli 1977), 25 (Holttum 1971, 1982, Old World only), 20 (Ching 1978), 5 (Smith 1990a, Smith et al. 2006, 2008), or only one (Morton 1969, Tryon and Tryon 1982).

Recent molecular phylogenetic studies have recovered two main groups in the family: the phegopteroid and the thelypteroid lineages (Smith and Cranfill 2002, He and Zhang 2012, and Almeida et al. 2015). The phegopteroid lineage includes two paleotropical genera, Macrothelypteris (H.Itô) Ching (*ca.* 10 spp.), Pseudophegopteris Ching (*ca.* 20 spp.), and one north-temperate genus, Phegopteris Fée (4 spp.). In the thelypteroid lineage, Thelypteris *s.s.* (Thelypteris palustris Schott, Thelypteris confluens (Thunb.) C.V.Morton) is the basal group and sister to a clade that includes all other non-phegopteroid Thelypteridaceae. This confirms the status of Thelypteris *s.s.* as a small, isolated segregate genus of two species. The sister group to Thelypteris *s.s.* comprises two well supported, much larger clades: the amauropeltoid clade, comprising the genera Amauropelta Kunze, Parathelypteris (H. Itô) Ching, Coryphopteris Holttum, and Metathelypteris (H.Itô) Ching, and the cyclosoroid clade, comprising several genera from the Old World and the New World (He and Zhang 2012, and Almeida et al. 2015). Inside the cylosoroid clade, several groups in the Paleotropics are still ill-defined and probably not monophyletic (He and Zhang 2012, Almeida et al. 2015). Thus, the current infrafamilial classification for Thelypteridaceae is not in accordance with growing phylogenetic evidence and principles of monophyly.

The following native Neotropical genera of Thelypteridaceae are recognized: Amauropelta, Christella Lév. (a more precise circumscription still pending), Cyclosorus Link, Goniopteris (C.Presl) Duek., Meniscium Schreber, Stegnogramma Blume, Steiropteris C.Chr., and Thelypteris Schmidel, based on the recent molecular phylogenetic data for this family (Almeida et al. 2015) and the morphological uniformity recognized in previously proposed classifications (Holttum 1971, Pichi Sermolli 1977, Smith 1980, 1990a). With the exception of Amauropelta and Thelypteris, the other six genera belong to the cyclosoroid clade. In the most recent published floras for Neotropical areas, these monophyletic groups of Thelypteridaceae were treated as subgenera of Thelypteris. In the most recent fern classifications (Smith 1990a; Smith et al. 2006, 2008; Christenhusz et al. 2011), Amauropelta was treated within the genus Thelypteris, and Christella, Cyclosorus, Goniopteris, Meniscium, and Steiropteris within the genus Cyclosorus.

Utilizing our most recent results on molecular phylogeny of the family (Almeida et al. 2015), the monophyletic genera accepted here have strong support. They are also easily defined morphologically by characters in the key below.

Amauropelta, predominantly Neotropical, is most closely related to several Old World genera: Coryphopteris, Metathelypteris, and Parathelypteris. Amauropelta is recognized mainly by the laminar base usually gradually or abruptly reduced with one to many pairs of auriculiform or glanduliform pinnae, and by the veins from adjacent segments usually meeting the blade margins above the sinuses on pinnatifid pinnae.

Our results (Almeida et al. 2015) confirm that Steiropteris is monophyletic and sister to all remaining cyclosoroid genera except Stegnogramma. These results include an interesting novelty: Thelypteris polypodioides (Raddi) C.F.Reed and Thelypteris villosa (Link) C.F.Reed, from southern Brazil (previously *incertae sedis*), nest in Steiropteris with strong support. These two species were treated by Christensen (1913) as belonging to Dryopteris subg. Leptogramma (J.Sm.) C.Chr. (= Stegnogramma).

Goniopteris has long been recognized as a natural group (Christensen 1913, Tryon and Tryon 1982, Smith 1990a). It is also monophyletic (Smith and Cranfill 2002, Schuettpelz and Pryer 2007) and has been accorded generic status, e.g., by Copeland (1947), Brade (1972) and Pichi Sermolli (1977). The presence of furcate or stellate hairs on the blades and/or rhizome apex scales is a useful diagnostic character for Goniopteris (Salino 2002), but this character has apparently been secondarily lost in a few species: Goniopteris mollis Fée (Mexico and Central America; Mickel and Smith 2004; Smith 1995a), Goniopteris macrotis (Hook.) Pic.Serm., Goniopteris semihastata (Kunze) Salino & T.E.Almeida (both endemic to Peru; Smith 1992), Goniopteris holodictya (K.U.Kramer) Salino & T.E.Almeida (French Guiana; Smith 1993), and Goniopteris clypeata (Maxon & C.V.Morton) Salino & T.E.Almeida (Panama, Peru; Smith 1995a, and Colombia).

The narrow circumscription of Cyclosorus used here has already been adopted by some authors (e.g., Pichi Sermolli 1977, Holttum 1971, 1982). This clade (pantropical, with two species, only one of which, Cyclosorus interruptus (Willd.) H.Itô, occurs in the Neotropics) is supported as monophyletic in the results of our molecular phylogeny (Almeida et al. 2015). Most native species previously considered in Thelypteris subg. Cyclosorus (Link) C.V.Morton in some floras (Mickel and Beitel 1988, Mickel and Smith 2004, Proctor 1977, 1985, 1989, Salino and Semir 2002, Smith 1981a,b, 1983, 1988, 1992, 1995a,b) are now treated in the genus Christella, as done by Pichi Sermolli (1977) and Holttum (1974, 1982). However, it appears likely that Christella sensu Holttum is not monophyletic (see Smith and Cranfill 2002; Schuettpelz and Pryer 2007; He and Zhang 2012).

Following Smith (1990a) and Smith et al. (2006, 2008), Mazumdar and Mukhopadhyay (2013) proposed new combinations in Cyclosorus for many species nested in the cyclosoroid clade. However, if this clade is to be considered as a single genus, the oldest generic name is Meniscium (Schreber 1791). In order to retain all the names recombined by Mazumdar and Mukhopadhyay (2013), Mazumdar (2015) subsequently proposed conservation of Cyclosorus against Meniscium. However, we believe conservation is unnecessary, because the cyclosoroid clade, considered by Mazumdar (2015) as a genus, corresponds to at least 10 genera according to recent molecular phylogenetic results (Almeida et al. 2015). We think it preferable that Cyclosorus *sensu stricto* be construed as comprising only two species (see above). At the same time, we interpret phylogenetic evidence to best support Meniscium as a genus comprising 26 species (Fernandes et al. 2014).

Meniscium, wholly Neotropical, appears to be monophyletic and more closely related to Cyclosorus *s.s.* (Cyclosorus interruptus), Mesophlebium Holttum, and Ampelopteris Kunze (Almeida et al. 2015). Meniscium is morphologically uniform and characterized by short-creeping rhizomes, pinnate leaves each with a conform apical pinna, and by regularly anastomosing veins resulting in parallel rows of areoles that include either a single free vein or a bisecting vein. Fernandes et al. (2014) made five new combinations for Meniscium species; combinations are already available for other recognized menisciums. Similarly, combinations are available for 38 species of Amauropelta, for nearly 40 species of Goniopteris, as well as for nine species of Steiropteris. New combinations in Amauropelta, Goniopteris, and Steiropteris are established for most remaining Neotropical species, those that appear commonly recognized in existing floras and monographs. We refrain from addressing further Christella sensu Holttum in the Neotropics, because (as mentioned above) existing evidence suggests that it is not monophyletic – most of the neotropical species (sect. Pelazoneuron Holttum) appear to comprise a clade separate from the paleotropical species (sect. Christella). However, sampling of Christella in both the Old World and New World, as well as affiliated cyclosoroid genera circumscribed by Holttum (1982) in the Paleotropics, is still far too meager to construct a viable taxonomy.

## Key to the native Neotropical Thelypteridaceae genera

**Table:** 

1	Forked or stellate hairs present at least on the rachises and costae, and/or on the scales at apices of rhizomes, and may be present in other parts of the frond	**Goniopteris**
–	All hairs unbranched (hairs fasciculate, i.e., basally clustered, in some species of Amauropelta)	
2	Laminae 1-pinnate, rarely simple; pinnae entire or with an serrate or undulate margin, rarely shallowly lobed; veins regularly anatomosing, areoles 3–25-seriate between costae and pinna margins	**3**
–	Laminae 1-pinnate-pinnatifid, rarely 2-pinnate; pinnae incised 1/3 their width or more; veins meeting the margins above sinuses, or connivent at sinuses, or with 1 or 2 pairs of anastomosing below sinuses	**4**
3	Sori usually in a single row between adjacent costules (except Meniscium andreanum, which often has a double row of sori between main lateral veins), oblong or linear, rarely round; sori exindusiate	**Meniscium**
–	Sori usually in a double row between adjacent costules, usually round (except Goniopteris holodictya, which sometimes has only one row of somewhat elongate sori between costules); sori indusiate (indusial may be small, or hidden in mature sori!) or exindusiate	**Goniopteris**
4	Sporangia setose; sori elongate, exindusiate	**5**
–	Sporangia not setose, or if setose (in eight species of Amauropelta) then sori round; sori round, oblong, or rarely elongate; sori indusiate or exindusiate	**6**
5	Pinnae broadly adnate and decurrent onto rachises in the distal half of laminae; proximal pinnae not cuneate at bases	**Stegnogramma (Stegnogramma pilosa, Stegnogramma burksiorum)**
–	Pinnae neither adnate nor decurrent onto rachises, sometimes one distal pair adnate to rachises; proximal pinnae short- to long-cuneate at bases	**Steiropteris (Stegnogramma polypodioides, Stegnogramma villosa)**
6	Laminae with all veins usually meeting margins above the sinuses	**7**
–	Laminae with at least some of the proximal veins running to the sinuses or forming an excurrent vein to the sinuses	**9**
7	Laminae narrowed proximally with (1-) 2−20 pairs of greatly reduced pinnae, the proximal pinnae usually auriculiform or glanduliform, rarely proximal pinnae not reduced	**Amauropelta**
–	Laminae usually not narrowed proximally, or only slightly so, usually lacking greatly reduced pinnae	**8**
8	Rhizomes long-creeping; laminae 1-pinnate-pinnatifid to 2-pinnate; veins of sterile laminae 1- or 2-forked, sometimes simple in the fertile laminae	**Thelypteris**
–	Stems ascending to erect, rarely short-creeping; laminae 1-pinnate-pinnatifid; veins of sterile and fertile laminae simple.. Steiropteris (subg. Glaphyropteris)
9	Rhizomes long-creeping to 3 m or more, blackish, scaleless or nearly so	**Cyclosorus**
–	Rhizomes short-creeping to ascending or erect, light brown, with conspicuous scales, at least at apices	**10**
10	Cartilaginous keel (false vein) present below pinna sinuses, keels extending toward costa but not meeting it; aerophores (tuberculate or often threadlike) at pinna bases usually present, absent in a few species; indusia present or absent	**Steiropteris (subg. Steiropteris)**
–	Cartilaginous keel absent below pinna sinuses; aerophores at pinna bases absent; indusia present, persistent	**Christella**

## New combinations

The names proposed as needing new combinations were obtained from taxonomic and floristic sources such as floras, monographs, or isolated published papers. All names were checked in IPNI.ORG and TROPICOS.ORG. The main floras consulted were ones for Mexico (Smith 1981a, 1988; Mickel and Beitel 1988, Mickel and Smith 2004), Lesser Antilles (Proctor 1977), Jamaica (Proctor 1985), Puerto Rico and the Virgin Islands (Proctor 1989), Cuba (Sánchez et al 2006), Mesoamerica (Smith 1995a), Guatemala (Smith 1981b), Venezuela (Vareschi 1969; Smith 1995b), Guianas (Smith 1993), Ecuador (Smith 1983), Peru (Smith 1992), Argentina (Ponce 1987), and from Brazil (Brade 1971, Sehnem 1979, Salino and Semir 2002, 2003, 2004, Ponce 2007, Salino and Almeida 2010). The monographs of reference were the works of Christensen (1907, 1913, 1920) and Smith (1980). In addition, the following works describing new species were consulted: Smith and Lellinger (1985), Smith (1990b), Salino (2002), Salino and Melo (2000), Smith and Kessler (2008), Matos et al. (2010), and Salino et al. (2011, 2014). The authorship of new combinations was established as follows: species in Venezuela are credited to Alan R. Smith; Alexandre Salino and Thais Elias Almeida are credited with authorship of the remaining species. We choose to cite only the basionym and the name in Thelypteris (when available) for all new combinations; this avoids citation of the often extensive synonymy, information that is available in many recent floristic and taxonomic studies.

### New combinations in Amauropelta

#### Amauropelta achalensis (Hieron.)

Taxon classificationPlantaePolypodialesThelypteridaceae

Salino & T.E.Almeida comb. nov.

urn:lsid:ipni.org:names:77151259-1

Aspidium achalense Hieron., Bot. Jahrb. Syst. 22: 371. 1896.
Thelypteris achalensis (Hieron.) Abbiatti, Darwiniana 13: 566. 1964.

#### Amauropelta aculeata (A.R.Sm.)

Taxon classificationPlantaePolypodialesThelypteridaceae

Salino & T.E.Almeida comb. nov.

urn:lsid:ipni.org:names:77150878-1

Thelypteris aculeata A.R.Sm., Fl. Ecuador 18: 15. F. 1A, 1B. 1983.

#### Amauropelta acunae (C.Sánchez & Zavaro)

Taxon classificationPlantaePolypodialesThelypteridaceae

Salino & T.E.Almeida comb. nov.

urn:lsid:ipni.org:names:77150879-1

Thelypteris acunae C.Sánchez & Zavaro, Fontqueria 31: 223. f.1. 1991.

#### Amauropelta aliena (C.Chr.)

Taxon classificationPlantaePolypodialesThelypteridaceae

Salino & T.E.Almeida comb. nov.

urn:lsid:ipni.org:names:77150880-1

Dryopteris aliena C.Chr., Kongl. Svenska Vetenskapsakad. Handl., ser. 3, 16: 23. 1937.

#### Amauropelta altitudinis (Ponce)

Taxon classificationPlantaePolypodialesThelypteridaceae

Salino & T.E.Almeida comb. nov.

urn:lsid:ipni.org:names:77151260-1

Thelypteris altitudinis Ponce, Darwiniana 28 (1-4): 345. 1987.

#### Amauropelta amambayensis (Ponce)

Taxon classificationPlantaePolypodialesThelypteridaceae

Salino & T.E.Almeida comb. nov.

urn:lsid:ipni.org:names:77151261-1

Thelypteris amambayensis Ponce, Candollea 55: 310.

#### Amauropelta amphioxypteris (Sodiro)

Taxon classificationPlantaePolypodialesThelypteridaceae

Salino & T.E.Almeida comb. nov.

urn:lsid:ipni.org:names:77150881-1

Nephrodium amphioxypteris Sodiro, Recens. Crypt. Vasc. Quit. 51. 1883.
Thelypteris amphioxypteris (Sodiro) A.R.Sm., Fl. Ecuador 18: 17. 1983.

#### Amauropelta andicola (A.R.Sm.)

Taxon classificationPlantaePolypodialesThelypteridaceae

Salino & T.E.Almeida comb. nov.

urn:lsid:ipni.org:names:77150882-1

Thelypteris andicola A.R.Sm., Fieldiana, Bot., n.s., 29: 16. 1992.

#### Amauropelta appressa (A.R.Sm.)

Taxon classificationPlantaePolypodialesThelypteridaceae

Salino & T.E.Almeida comb. nov.

urn:lsid:ipni.org:names:77150883-1

Thelypteris appressa A.R.Sm., Fl. Ecuador 18: 19. 1983.

#### Amauropelta araucariensis (Ponce)

Taxon classificationPlantaePolypodialesThelypteridaceae

Salino & T.E.Almeida comb. nov.

urn:lsid:ipni.org:names:77150884-1

Thelypteris araucariensis Ponce, Darwiniana 33: 270. 1995.

#### Amauropelta arborea (Brause)

Taxon classificationPlantaePolypodialesThelypteridaceae

A.R.Sm. comb. nov.

urn:lsid:ipni.org:names:77151262-1

Dryopteris arborea Brause, Repert. Spec. Nov. Regni Veg. 13: 294. 1914.
Thelypteris arborea (Brause) A.R.Sm., Acta Bot. Venez. 14 (3): 7. 1984.

#### Amauropelta arenosa (A.R.Sm.)

Taxon classificationPlantaePolypodialesThelypteridaceae

A.R.Sm. comb. nov.

urn:lsid:ipni.org:names:77150885-1

Thelypteris arenosa A.R.Sm., Fl. Ecuador 18: 21. 1983.

#### Amauropelta argentina (Hieron.)

Taxon classificationPlantaePolypodialesThelypteridaceae

Salino & T.E.Almeida comb. nov.

urn:lsid:ipni.org:names:77151263-1

Aspidium argentinum Hieron., Bot. Jahrb. Syst. 22: 367. 1896.
Thelypteris argentina (Hieron.) Abbiatti, Revista Mus. La Plata, Secc. Bot. 9 (36-37): 19. 1958.

#### Amauropelta arrecta (A.R.Sm.)

Taxon classificationPlantaePolypodialesThelypteridaceae

Salino & T.E.Almeida comb. nov.

urn:lsid:ipni.org:names:77150886-1

Thelypteris arrecta A.R.Sm., Fieldiana, Bot., n.s., 29: 36. 1992.

#### Amauropelta atrorubens (Mett. ex Kuhn)

Taxon classificationPlantaePolypodialesThelypteridaceae

Salino & T.E.Almeida comb. nov.

urn:lsid:ipni.org:names:77151264-1

Aspidium atrorubens Mett. ex Kuhn, Linnaea 36. 112. 1869.
Thelypteris atrorubens (Mett. ex Kuhn) A.R.Sm., Fieldiana, Bot., n.s. 29: 24. 1992.

#### Amauropelta atrovirens (C.Chr.)

Taxon classificationPlantaePolypodialesThelypteridaceae

Salino & T.E.Almeida comb. nov.

urn:lsid:ipni.org:names:77150887-1

Dryopteris atrovirens C.Chr., Bull. Herb. Boissier, sér. 2, 7 (4): 263. 1907.
Thelypteris atrovirens (C.Chr.) C.F.Reed, Phytologia 17 (4): 261. 1968.

#### Amauropelta aymarae (A.R.Sm. & M.Kessler)

Taxon classificationPlantaePolypodialesThelypteridaceae

Salino & T.E.Almeida comb. nov.

urn:lsid:ipni.org:names:77150888-1

Thelypteris aymarae A.R.Sm. & M.Kessler, Brittonia 60 (1): 49. 2008.

#### Amauropelta balbisii (Spreng.)

Taxon classificationPlantaePolypodialesThelypteridaceae

A.R.Sm. comb. nov.

urn:lsid:ipni.org:names:77150889-1

Polypodium balbisii Spreng., Nova Acta Phys.-Med. Acad. Caes. Leop.-Carol. Nat. Cur. 10: 228. 1821.
Thelypteris balbisii (Spreng.) Ching, Bull. Fan Mem. Inst. Biol. 10: 250. 1941.

#### Amauropelta barvae (A.R.Sm.)

Taxon classificationPlantaePolypodialesThelypteridaceae

Salino & T.E.Almeida comb. nov.

urn:lsid:ipni.org:names:77150890-1

Thelypteris barvae A.R.Sm., Ann. Missouri Bot. Gard. 77 (1): 118. f. 2A-C. 1990.

#### Amauropelta basisceletica (C.Sánchez, Caluff & O.Alvarez)

Taxon classificationPlantaePolypodialesThelypteridaceae

Salino & T.E.Almeida comb. nov.

urn:lsid:ipni.org:names:77151265-1

Thelypteris basisceletica C.Sánchez, Caluff & O.Alvarez, Amer. Fern J. 95 (1): 30. f. 1. 2005.

#### Amauropelta binervata (A.R.Sm.)

Taxon classificationPlantaePolypodialesThelypteridaceae

A.R.Sm. comb. nov.

urn:lsid:ipni.org:names:77150891-1

Thelypteris binervata A.R.Sm., Acta Bot. Venez. 14 (3): 6. 1984.

#### Amauropelta bonapartii (Rosenst.)

Taxon classificationPlantaePolypodialesThelypteridaceae

Salino & T.E.Almeida comb. nov.

urn:lsid:ipni.org:names:77151266-1

Dryopteris bonapartii Rosenst., Repert. Spec. Nov. Regni Veg. 7: 303–304. 1909.
Thelypteris bonapartii (Rosenst.) Alston, J. Wash. Acad. Sci. 48: 233.1958.

#### Amauropelta brachypoda (Baker)

Taxon classificationPlantaePolypodialesThelypteridaceae

A.R.Sm. comb. nov.

urn:lsid:ipni.org:names:77150892-1

Nephrodium brachypodum Baker, Timehri 5: 213. 1886.
Thelypteris brachypoda (Baker) C.V.Morton, Fieldiana, Bot. 28: 10. 1951.

#### Amauropelta brachypus (Sodiro)

Taxon classificationPlantaePolypodialesThelypteridaceae

Salino & T.E.Almeida comb. nov.

urn:lsid:ipni.org:names:77150893-1

Nephrodium brachypus Sodiro, Recens. Crypt. Vasc. Quit. 43. 1883.
Thelypteris brachypus (Sodiro) R.M.Tryon & A.F.Tryon, Rhodora 84: 128. 1982.

#### Amauropelta brausei (Hieron.)

Taxon classificationPlantaePolypodialesThelypteridaceae

A.R.Sm. comb. nov.

urn:lsid:ipni.org:names:77150894-1

Dryopteris brausei Hieron., Hedwigia 46: 337, t. 6, f. 11. 1907.
Thelypteris brausei (Hieron.) Alston, J. Wash. Acad. Sci. 48: 233. 1958.

#### Amauropelta burkartii (Abbiatti)

Taxon classificationPlantaePolypodialesThelypteridaceae

Salino & T.E.Almeida comb. nov.

urn:lsid:ipni.org:names:77150895-1

Thelypteris burkartii Abbiatti, Darwiniana 13: 550, f. 4, t. 2. 1964.

#### Amauropelta campii (A.R.Sm.)

Taxon classificationPlantaePolypodialesThelypteridaceae

Salino & T.E.Almeida comb. nov.

urn:lsid:ipni.org:names:77151267-1

Thelypteris campii A.R.Sm., Fl. Ecuador 18: 27. 1983.

#### Amauropelta canadasii (Sodiro)

Taxon classificationPlantaePolypodialesThelypteridaceae

Salino & T.E.Almeida comb. nov.

urn:lsid:ipni.org:names:77150896-1

Nephrodium canadasii Sodiro, Recens. Crypt. Vasc. Quit. 48. 1883.
Thelypteris canadasii (Sodiro) Alston, J. Wash. Acad. Sci 48: 234. 1958.

#### Amauropelta caucaensis (Hieron.)

Taxon classificationPlantaePolypodialesThelypteridaceae

A.R.Sm. comb. nov.

urn:lsid:ipni.org:names:77151268-1

Nephrodium caucaense Hieron., Bot. Jahrb. Syst. 34: 444. 1904.
Thelypetris caucaensis (Hieron.) Alston, J. Wash. Acad. Sci 48: 233. 1958.

#### Amauropelta chaparensis (A.R.Sm. & M.Kessler)

Taxon classificationPlantaePolypodialesThelypteridaceae

Salino & T.E.Almeida comb. nov.

urn:lsid:ipni.org:names:77150897-1

Thelypteris chaparensis A.R.Sm. & M.Kessler, Brittonia 60 (1): 51. 2008.

#### Amauropelta chiriquiana (A.R.Sm.)

Taxon classificationPlantaePolypodialesThelypteridaceae

Salino & T.E.Almeida comb. nov.

urn:lsid:ipni.org:names:77150898-1

Thelypteris chiriquiana A.R.Sm., Ann. Missouri Bot. Gard. 77 (1): 120. f. 3A-C. 1990.

#### Amauropelta christensenii (Christ ex C.Chr.)

Taxon classificationPlantaePolypodialesThelypteridaceae

Salino & T.E.Almeida comb. nov.

urn:lsid:ipni.org:names:77151269-1

Dryopteris christensenii Christ ex C.Chr., Bull. Herb. Boissier, sér. 2, 7 (4): 263. 1907.
Thelypteris christensenii (Christ ex C.Chr.) C.F.Reed, Phytologia 17 (4): 267. 1968.

#### Amauropelta cinerea (Sodiro)

Taxon classificationPlantaePolypodialesThelypteridaceae

A.R.Sm. comb. nov.

urn:lsid:ipni.org:names:77150900-1

Nephrodium cinereum Sodiro, Sert. Fl. Ecuad. II. 26. 1908.
Thelypteris cinerea (Sodiro) A.R.Sm., Phytologia 34: 233. 1976.

#### Amauropelta cochaensis (C.Chr.)

Taxon classificationPlantaePolypodialesThelypteridaceae

Salino & T.E.Almeida comb. nov.

urn:lsid:ipni.org:names:77150901-1

Dryopteris cochaensis C.Chr., Kongel. Danske Vidensk. Selsk. Skr., Naturvidensk. Math. Afd., ser. 7, 10: 152. 1913.

#### Amauropelta cocos (A.R.Sm. & Lellinger)

Taxon classificationPlantaePolypodialesThelypteridaceae

Salino & T.E.Almeida comb. nov.

urn:lsid:ipni.org:names:77150904-1

Thelypteris cocos A.R.Sm. & Lellinger, Proc. Biol. Soc. Wash. 98 (4): 918. f. 3. 1985.

#### Amauropelta comptula (A.R.Sm.)

Taxon classificationPlantaePolypodialesThelypteridaceae

Salino & T.E.Almeida comb. nov.

urn:lsid:ipni.org:names:77150906-1

Thelypteris comptula A.R.Sm., Fieldiana, Bot., n.s., 29: 23. 1992.

#### Amauropelta conformis (Sodiro)

Taxon classificationPlantaePolypodialesThelypteridaceae

Salino & T.E.Almeida comb. nov.

urn:lsid:ipni.org:names:77150908-1

Nephrodium conforme Sodiro, Recens. Crypt. Vasc. Quit. 45. 1883.
Thelypteris conformis (Sodiro) A.R.Sm., Fl. Ecuador 18: 37. 1983.

#### Amauropelta consanguinea (Fée)

Taxon classificationPlantaePolypodialesThelypteridaceae

Salino & T.E.Almeida comb. nov.

urn:lsid:ipni.org:names:77150910-1

Aspidium consanguineum Fée, Mém. Foug. 11 (Hist. Foug. Ant.): 76, t. 20, f. 3). 1866.
Thelypteris consanguinea (Fée) Proctor, Rhodora 61 (732): 306. 1959.

#### Amauropelta cooleyi (Proctor)

Taxon classificationPlantaePolypodialesThelypteridaceae

Salino & T.E.Almeida comb. nov.

urn:lsid:ipni.org:names:77150912-1

Thelypteris cooleyi Proctor, Rhodora 68: 468. 1966.

#### Amauropelta corazonensis (Baker)

Taxon classificationPlantaePolypodialesThelypteridaceae

Salino & T.E.Almeida comb. nov.

urn:lsid:ipni.org:names:77151270-1

Nephrodium corazonense Baker, J. Bot. 15: 163. 1877, as carazanensis”.
Thelypteris corazonensis (Baker) A.R.Sm., Fl. Ecuador 18: 38. 1983.

#### Amauropelta cornuta (Maxon)

Taxon classificationPlantaePolypodialesThelypteridaceae

Salino & T.E.Almeida comb. nov.

urn:lsid:ipni.org:names:77150914-1

Dryopteris cornuta Maxon, J. Wash. Acad. Sci. 19 (12): 245. f. 1. 1929.
Thelypteris cornuta (Maxon) Ching, Bull. Fan Mem. Inst. Biol. 10: 251. 1941.

#### Amauropelta correllii (A.R.Sm.)

Taxon classificationPlantaePolypodialesThelypteridaceae

Salino & T.E.Almeida comb. nov.

urn:lsid:ipni.org:names:77150916-1

Thelypteris correllii A.R.Sm., Fl. Ecuador 18: 39. 1983.

#### Amauropelta crassiuscula (C.Chr. & Maxon)

Taxon classificationPlantaePolypodialesThelypteridaceae

Salino & T.E.Almeida comb. nov.

urn:lsid:ipni.org:names:77150917-1

Dryopteris crassiuscula C.Chr. & Maxon, Amer. Fern J. 23: 75. 1933.
Thelypteris crassiuscula (C.Chr. & Maxon) Lellinger, Amer. Fern J. 67(2): 60. 1977.

#### Amauropelta ctenitoides (A.R.Sm.)

Taxon classificationPlantaePolypodialesThelypteridaceae

Salino & T.E.Almeida comb. nov.

urn:lsid:ipni.org:names:77150920-1

Thelypteris ctenitoides A.R.Sm., Fieldiana, Bot., n.s., 29: 37. 1992.

#### Amauropelta decrescens (Proctor)

Taxon classificationPlantaePolypodialesThelypteridaceae

Salino & T.E.Almeida comb. nov.

urn:lsid:ipni.org:names:77151271-1

Thelypteris decrescens Proctor, Amer. Fern J. 71(2): 57. 1981.

#### Amauropelta decurtata (Link)

Taxon classificationPlantaePolypodialesThelypteridaceae

Salino & T.E.Almeida comb. nov.

urn:lsid:ipni.org:names:77151422-1

Asplenium decurtatum Link Fil. sp. 94. 1841.

#### Amauropelta deflectens (C.Chr.)

Taxon classificationPlantaePolypodialesThelypteridaceae

Salino & T.E.Almeida comb. nov.

urn:lsid:ipni.org:names:77150922-1

Dryopteris deflectens C.Chr., Kongl. Svenska Vetenskapsakad. Handl., ser. 3, 16(2): 25, t. 3, f. 9-11. 1937.

#### Amauropelta delasotae (A.R.Sm. & Lellinger)

Taxon classificationPlantaePolypodialesThelypteridaceae

Salino & T.E.Almeida comb. nov.

urn:lsid:ipni.org:names:77150924-1

Thelypteris delasotae A.R.Sm. & Lellinger, Proc. Biol. Soc. Wash. 98(4): 919. f. 5. 1985.

#### Amauropelta demissa (A.R.Sm.)

Taxon classificationPlantaePolypodialesThelypteridaceae

Salino & T.E.Almeida comb. nov.

urn:lsid:ipni.org:names:77150926-1

Thelypteris demissa A.R.Sm., Fieldiana, Bot., n.s., 29: 21. 1992.

#### Amauropelta denudata (C.Sánchez & Caluff)

Taxon classificationPlantaePolypodialesThelypteridaceae

Salino & T.E.Almeida comb. nov.

urn:lsid:ipni.org:names:77150928-1

Thelypteris denudata C.Sánchez & Caluff, Willdenowia 35: 159. 2005.

#### Amauropelta dodsonii (A.R.Sm.)

Taxon classificationPlantaePolypodialesThelypteridaceae

Salino & T.E.Almeida comb. nov.

urn:lsid:ipni.org:names:77150930-1

Thelypteris dodsonii A.R.Sm., Fl. Ecuador 18: 41. 1983.

#### Amauropelta dudleyi (A.R.Sm.)

Taxon classificationPlantaePolypodialesThelypteridaceae

Salino & T.E.Almeida comb. nov.

urn:lsid:ipni.org:names:77150932-1

Thelypteris dudleyi A.R.Sm., Fieldiana, Bot., n.s., 29: 33. 1992.

#### Amauropelta elegantula (Sodiro)

Taxon classificationPlantaePolypodialesThelypteridaceae

Salino & T.E.Almeida comb. nov.

urn:lsid:ipni.org:names:77150933-1

Nephrodium elegantulum Sodiro, Crypt. Vasc. Quit. 243. 1893.
Thelypteris elegantula (Sodiro) Alston, J. Wash. Acad. Sci. 48: 233. 1958.

#### Amauropelta enigmatica (A.R.Sm.)

Taxon classificationPlantaePolypodialesThelypteridaceae

Salino & T.E.Almeida comb. nov.

urn:lsid:ipni.org:names:77150936-1

Thelypteris enigmatica A.R.Sm., Fieldiana, Bot., n.s., 29: 16. 1992.

#### Amauropelta eriosorus (Fée)

Taxon classificationPlantaePolypodialesThelypteridaceae

Salino & T.E.Almeida comb. nov.

urn:lsid:ipni.org:names:77151278-1

Aspidium eriosorus Fée, Crypt. Vasc. Brésil, 2. 73. T. 101. 1873.
Thelypteris eriosorus (Fée) Ponce (as *T. eriosora*), Novon 8: 275. 1998.

#### Amauropelta euchlora (Sodiro)

Taxon classificationPlantaePolypodialesThelypteridaceae

A.R.Sm. comb. nov.

urn:lsid:ipni.org:names:77150938-1

Polypodium euchlorum Sodiro, Recens. Crypt. Vasc. Quit. 58. 1883.
Thelypteris euchlora (Sodiro) C.F.Reed, Phytologia 17(4): 275. 1968.

#### Amauropelta euthythrix (A.R.Sm.)

Taxon classificationPlantaePolypodialesThelypteridaceae

Salino & T.E.Almeida comb. nov.

urn:lsid:ipni.org:names:77150940-1

Thelypteris euthythrix A.R.Sm., Fl. Ecuador 18: 44. 1983.

#### Amauropelta exuta (A.R.Sm.)

Taxon classificationPlantaePolypodialesThelypteridaceae

Salino & T.E.Almeida comb. nov.

urn:lsid:ipni.org:names:77151279-1

Thelypteris exuta A.R.Sm., Fl. Ecuador 18: 44. 1983.

#### Amauropelta fasciola (A.R.Sm. & M.Kessler)

Taxon classificationPlantaePolypodialesThelypteridaceae

Salino & T.E.Almeida comb. nov.

urn:lsid:ipni.org:names:77150942-1

Thelypteris fasciola A.R.Sm. & M.Kessler, Brittonia 60 (1): 51. 2008.

#### Amauropelta fayorum (A.R.Sm. & M.Kessler)

Taxon classificationPlantaePolypodialesThelypteridaceae

Salino & T.E.Almeida comb. nov.

urn:lsid:ipni.org:names:77150944-1

Thelypteris fayorum A.R.Sm. & M.Kessler, Brittonia 60(1): 53. 2008.

#### Amauropelta firma (Baker ex Jenman)

Taxon classificationPlantaePolypodialesThelypteridaceae

Salino & T.E.Almeida comb. nov.

urn:lsid:ipni.org:names:77150946-1

Nephrodium firmum Baker ex Jenman., J. Bot. 17: 260. 1879.
Thelypteris firma (Baker ex Jenman) Proctor, Bull. Inst. Jamaica, Sci. Ser. 5: 60. 1953.

#### Amauropelta fluminalis (A.R.Sm.)

Taxon classificationPlantaePolypodialesThelypteridaceae

Salino & T.E.Almeida comb. nov.

urn:lsid:ipni.org:names:77150948-1

Thelypteris fluminalis A.R.Sm., Fl. Ecuador 18: 46. 1983.

#### Amauropelta frigida (Christ)

Taxon classificationPlantaePolypodialesThelypteridaceae

A.R.Sm. comb. nov.

urn:lsid:ipni.org:names:77150949-1

Aspidium frigidum Christ, Bull. Herb. Boissier, sér. 2., 6: 160. 1906.
Thelypteris frigida (Christ) A.R.Sm. & Lellinger, Amer. Fern J. 75(1): 31. 1985.

#### Amauropelta funckii (Mett.)

Taxon classificationPlantaePolypodialesThelypteridaceae

A.R.Sm. comb. nov.

urn:lsid:ipni.org:names:77150952-1

Aspidium funckii Mett., Ann. Sci. Nat., Bot., sér. 5, 2: 246. 1864.
Thelypteris funckii (Mett.) Alston, J. Wash. Acad. Sci. 48: 233. 1958.

#### Amauropelta furfuracea (A.R.Sm.)

Taxon classificationPlantaePolypodialesThelypteridaceae

Salino & T.E.Almeida comb. nov.

urn:lsid:ipni.org:names:77150954-1

Thelypteris furfuracea A.R.Sm., Fieldiana, Bot., n.s., 29: 34. 1992.

#### Amauropelta furva (Maxon)

Taxon classificationPlantaePolypodialesThelypteridaceae

Salino & T.E.Almeida comb. nov.

urn:lsid:ipni.org:names:77150956-1

Dryopteris furva Maxon, J. Wash. Acad. Sci. 34: 24. 1944.
Thelypteris furva (Maxon) R.M.Tryon, Rhodora 69: 6. 1977.

#### Amauropelta germaniana (Fée)

Taxon classificationPlantaePolypodialesThelypteridaceae

Salino & T.E.Almeida comb. nov.

urn:lsid:ipni.org:names:77150958-1

Phegopteris germaniana Fée, Mém. Foug. 11 (Hist. Foug. Ant.): 55. 1866.
Thelypteris germaniana (Fée) Proctor, Rhodora 61: 306. 1959.

#### Amauropelta glandulosolanosa (C.Chr.)

Taxon classificationPlantaePolypodialesThelypteridaceae

Salino & T.E.Almeida comb. nov.

urn:lsid:ipni.org:names:77150960-1

Dryopteris glandulosolanosa C.Chr., Dansk Bot. Ark. 9(3): 61. 1937.
Thelypteris glandulosolanosa (C.Chr.) R.M.Tryon, Rhodora 69: 6. 1967.

#### Amauropelta glutinosa (C.Chr.)

Taxon classificationPlantaePolypodialesThelypteridaceae

Salino & T.E.Almeida comb. nov.

urn:lsid:ipni.org:names:77150962-1

Dryopteris glutinosa C.Chr., Kongl. Svenska Vetenskapsakad. Handl., ser. 3, 16(2): 18. 1937.
Thelypteris glutinosa (C.Chr.) C.V.Morton, Amer. Fern J. 53(2): 66. 1963.

#### Amauropelta gomeziana (A.R.Sm. & Lellinger)

Taxon classificationPlantaePolypodialesThelypteridaceae

Salino & T.E.Almeida comb. nov.

urn:lsid:ipni.org:names:77150964-1

Thelypteris gomeziana A.R.Sm. & Lellinger, Proc. Biol. Soc. Wash. 98(4): 921. f 6. 1985.

#### Amauropelta gracilenta (Jenman)

Taxon classificationPlantaePolypodialesThelypteridaceae

Salino & T.E.Almeida comb. nov.

urn:lsid:ipni.org:names:77150965-1

Polypodium gracilentum Jenman, Bull. Bot. Dept. Jamaica 4. 129. 1897.
Thelypteris gracilenta (Jenman) Proctor, Amer. Fern J. 71(2): 60. 1981.

#### Amauropelta gracilis (Heward)

Taxon classificationPlantaePolypodialesThelypteridaceae

A.R.Sm. comb. nov.

urn:lsid:ipni.org:names:77150968-1

Gymnogramma gracilis Heward, Mag. Nat. Hist., n.s, 2: 457. 1838.
Thelypteris gracilis (Heward) Proctor, Bull. Inst. Jamaica, Sci. Ser. 5: 60. 1953.

#### Amauropelta grayumii (A.R.Sm.)

Taxon classificationPlantaePolypodialesThelypteridaceae

Salino & T.E.Almeida comb. nov.

urn:lsid:ipni.org:names:77150970-1

Thelypteris grayumii A.R.Sm., Ann. Missouri Bot. Gard. 77(1): 122. f 2D-E. 1990.

#### Amauropelta harrisii (Proctor)

Taxon classificationPlantaePolypodialesThelypteridaceae

Salino & T.E.Almeida comb. nov.

urn:lsid:ipni.org:names:77151280-1

Thelypteris harrisii Proctor, Amer. Fern J. 71: 59. 1981.

#### Amauropelta hastiloba (C.Chr.)

Taxon classificationPlantaePolypodialesThelypteridaceae

Salino & T.E.Almeida comb. nov.

urn:lsid:ipni.org:names:77150972-1

Dryopteris hastiloba C.Chr., Kongl. Svenska Vetenskapsakad. Handl., ser. 3, 16(2): 20. t 4, f 4-5. 1937.

#### Amauropelta heineri (C.Chr.)

Taxon classificationPlantaePolypodialesThelypteridaceae

Salino & T.E.Almeida comb. nov.

urn:lsid:ipni.org:names:77150974-1

Dryopteris heineri C.Chr., Repert. Spec. Nov. Regni Veg. 6: 380. 1909.
Thelypteris heineri (C.Chr.) C.F.Reed, Phytologia 17(4): 282. 1968.

#### Amauropelta hutchisonii (A.R.Sm.)

Taxon classificationPlantaePolypodialesThelypteridaceae

Salino & T.E.Almeida comb. nov.

urn:lsid:ipni.org:names:77150976-1

Thelypteris hutchisonii A.R.Sm., Fieldiana, Bot., n.s., 29: 29. 1992.

#### Amauropelta hydrophila (Fée)

Taxon classificationPlantaePolypodialesThelypteridaceae

Salino & T.E.Almeida comb. nov.

urn:lsid:ipni.org:names:77150978-1

Phegopteris hydrophila Fée, Mém. Foug. 11 (Hist. Foug. Ant.): 56. pl 13, f 3. 1866.
Thelypteris hydrophila (Fée) Proctor, Rhodora 61: 306. 1959.

#### Amauropelta illicita (Christ)

Taxon classificationPlantaePolypodialesThelypteridaceae

Salino & T.E.Almeida comb. nov.

urn:lsid:ipni.org:names:77150980-1

Dryopteris illicita Christ, Bull. Soc. Bot. Genève sér. 2, 1(5): 225. 1909.
Thelypteris illicita (Christ) C.F.Reed, Phytologia 17(4) : 284. 1968.

#### Amauropelta inabonensis (Proctor)

Taxon classificationPlantaePolypodialesThelypteridaceae

Salino & T.E.Almeida comb. nov.

urn:lsid:ipni.org:names:77151281-1

Thelypteris inabonensis Proctor, Amer. Fern J. 75(2): 61. f 3. 1985.

#### Amauropelta inaequans (C.Chr.)

Taxon classificationPlantaePolypodialesThelypteridaceae

Salino & T.E.Almeida comb. nov.

urn:lsid:ipni.org:names:77150981-1

Dryopteris euchlora (Sodiro) C.Chr. var. inaequans C.Chr., Kongel. Danske Vidensk. Selsk. Skr., Naturvidensk. Math. Afd., ser. 7, 10: 150. 1913.
Thelypteris inaequans (C.Chr.) Lellinger, Amer. Fern J. 67(2): 60. 1977.

#### Amauropelta inaequilateralis (A.R.Sm. & M.Kessler)

Taxon classificationPlantaePolypodialesThelypteridaceae

Salino & T.E.Almeida comb. nov.

urn:lsid:ipni.org:names:77150984-1

Thelypteris inaequilateralis A.R.Sm. & M.Kessler, Brittonia 60(1): 53. 2008.

#### Amauropelta insignis (Mett.)

Taxon classificationPlantaePolypodialesThelypteridaceae

Salino & T.E.Almeida comb. nov.

urn:lsid:ipni.org:names:77151282-1

Aspidium insigne Mett., Ann. Sci. Nat., Bot., sér. 5, 2: 247. 1864.
Thelypteris insignis (Mett.) Ching, Bull. Fan Mem. Inst. Biol. 10: 242. 1941.

#### Amauropelta intromissa (C.Chr.)

Taxon classificationPlantaePolypodialesThelypteridaceae

Salino & T.E.Almeida comb. nov.

urn:lsid:ipni.org:names:77150986-1

Dryopteris intromissa C.Chr., Kungl. Svenska Vetenskapsakad. Handl., ser. 3., 16(2): 22. t 4, f 9-10. 1937.

#### Amauropelta ireneae (Brade)

Taxon classificationPlantaePolypodialesThelypteridaceae

Salino & T.E.Almeida comb. nov.

urn:lsid:ipni.org:names:77150988-1

Dryopteris ireneae Brade, Sellowia 17: 57. f 4. 1965.
Thelypteris ireneae (Brade) Lellinger, Amer. Fern. J. 74(2): 60. 1984.

#### Amauropelta jimenezii (Maxon & C.Chr.)

Taxon classificationPlantaePolypodialesThelypteridaceae

Salino & T.E.Almeida comb. nov.

urn:lsid:ipni.org:names:77151283-1

Dryopteris jimenezii Maxon & C.Chr., Amer. Fern J. 4: 79. 1914.
Thelypteris jimenezii (Maxon & C.Chr.) C.F.Reed, Phytologia 17(4): 285. 1968.

#### Amauropelta juergensii (Rosenst.)

Taxon classificationPlantaePolypodialesThelypteridaceae

Salino & T.E.Almeida comb. nov.

urn:lsid:ipni.org:names:77150990-1

Nephrodium juergensii Rosenst., Festschrift Albert von Bamberg 63. 1905.
Thelypteris juergensii (Rosenst.) C.F.Reed, Phytologia 17(4): 286. 1968.

#### Amauropelta jujuyensis (de la Sota)

Taxon classificationPlantaePolypodialesThelypteridaceae

Salino & T.E.Almeida comb. nov.

urn:lsid:ipni.org:names:77150992-1

Thelypteris jujuyensis de la Sota, Darwiniana 18: 222. f 3, 8c. 1973.

#### Amauropelta laevigata (Mett. ex Kuhn)

Taxon classificationPlantaePolypodialesThelypteridaceae

Salino & T.E.Almeida comb. nov.

urn:lsid:ipni.org:names:77150994-1

Phegopteris laevigata Mett. ex Kuhn, Linnaea 36. 112. 1869.
Thelypteris laevigata (Mett. ex Kuhn) R.M.Tryon, Rhodora 69: 6. 1967.

#### Amauropelta leoniae (A.R.Sm.)

Taxon classificationPlantaePolypodialesThelypteridaceae

Salino & T.E.Almeida comb. nov.

urn:lsid:ipni.org:names:77150996-1

Thelypteris leoniae A.R.Sm., Fieldiana, Bot., n.s., 29: 18. 1992.

#### Amauropelta lepidula (Hieron.)

Taxon classificationPlantaePolypodialesThelypteridaceae

A.R.Sm. comb. nov.

urn:lsid:ipni.org:names:77150997-1

Dryopteris lepidula Hieron., Hedwigia 46. 328, t. 4, f. 4. 1907.
Thelypteris lepidula (Hieron.) Alston, J. Wash. Acad. Sci. 48: 233. 1958.

#### Amauropelta longicaulis (Baker)

Taxon classificationPlantaePolypodialesThelypteridaceae

Salino & T.E.Almeida comb. nov.

urn:lsid:ipni.org:names:77151000-1

Nephrodium longicaule Baker, J. Bot. 19: 204. 1881.
Thelypteris longicaulis (Baker) C.F.Reed, Phytologia 17(4): 289. 1968.

#### Amauropelta longipilosa (Sodiro)

Taxon classificationPlantaePolypodialesThelypteridaceae

Salino & T.E.Almeida comb. nov.

urn:lsid:ipni.org:names:77151284-1

Nephrodium longipilosum Sodiro, Anales Univ. Central Quito Ecuador 22(160): 103. 1908.
Thelypteris longipilosa (Sodiro) C.F.Reed, Phytologia 17(4): 290. 1968.

#### Amauropelta longisora (A.R.Sm.)

Taxon classificationPlantaePolypodialesThelypteridaceae

Salino & T.E.Almeida comb. nov.

urn:lsid:ipni.org:names:77151002-1

Thelypteris longisora A.R.Sm., Ann. Missouri Bot. Gard. 77(1): 123. f 3D-E. 1990.

#### Amauropelta loreae (A.R.Sm.)

Taxon classificationPlantaePolypodialesThelypteridaceae

Salino & T.E.Almeida comb. nov.

urn:lsid:ipni.org:names:77151003-1

Thelypteris loreae A.R.Sm., Mem. New York Bot. Gard. 88: 629–630. f 303A-E, 2004.

#### Amauropelta loretensis (A.R.Sm.)

Taxon classificationPlantaePolypodialesThelypteridaceae

Salino & T.E.Almeida comb. nov.

urn:lsid:ipni.org:names:77151006-1

Thelypteris loretensis A.R.Sm., Fieldiana, Bot., n.s., 29: 30. 1992.

#### Amauropelta lumbricoides (A.R.Sm. & M.Kessler)

Taxon classificationPlantaePolypodialesThelypteridaceae

Salino & T.E.Almeida comb. nov.

urn:lsid:ipni.org:names:77151008-1

Thelypteris lumbricoides A.R.Sm. & M.Kessler, Brittonia 60(1): 54. 2008.

#### Amauropelta macra (A.R.Sm.)

Taxon classificationPlantaePolypodialesThelypteridaceae

Salino & T.E.Almeida comb. nov.

urn:lsid:ipni.org:names:77151010-1

Thelypteris macra A.R.Sm., Fl. Ecuador 18: 54. 1983.

#### Amauropelta madidiensis (A.R.Sm. & M.Kessler)

Taxon classificationPlantaePolypodialesThelypteridaceae

Salino & T.E.Almeida comb. nov.

urn:lsid:ipni.org:names:77151012-1

Thelypteris madidiensis A.R.Sm. & M.Kessler, Brittonia 60(1): 54. 2008.

#### Amauropelta malangae (C.Chr.)

Taxon classificationPlantaePolypodialesThelypteridaceae

Salino & T.E.Almeida comb. nov.

urn:lsid:ipni.org:names:77151014-1

Dryopteris malangae C.Chr., Kongl. Svenska Vetenskapsakad. Handl., ser. 3, 16(2): 21. t. 4(6−8). 1937.
Thelypteris malangae (C.Chr.) C.V.Morton, Amer. Fern J. 53(2): 66. 1963.

#### Amauropelta melanochlaena (C.Chr.)

Taxon classificationPlantaePolypodialesThelypteridaceae

Salino & T.E.Almeida comb. nov.

urn:lsid:ipni.org:names:77151285-1

Dryopteris melanochlaena C.Chr., Smithsonian Misc. Collect. 52(3): 384. 1909.
Thelypteris melanochlaena (C.Chr.) C.F.Reed, Phytologia 17(4): 292. 1968.

#### Amauropelta mertensioides (C.Chr.)

Taxon classificationPlantaePolypodialesThelypteridaceae

Salino & T.E.Almeida comb. nov.

urn:lsid:ipni.org:names:77151016-1

Dryopteris mertensioides C.Chr., Kongel. Danske.Vidensk. Selsk. Skr., Naturvidensk. Math. Afd., ser. 7, 4: 328, f. 50. 1907.
Thelypteris mertensioides (C.Chr.) C.F.Reed, Phytologia 17(4): 292. 1968.

#### Amauropelta metteniana (Ching)

Taxon classificationPlantaePolypodialesThelypteridaceae

Salino & T.E.Almeida comb. nov.

urn:lsid:ipni.org:names:77151017-1

Thelypteris metteniana Ching, Bull. Fan mem. Inst. Biol. 10: 252. 1941.

#### Amauropelta micula (A.R.Sm.)

Taxon classificationPlantaePolypodialesThelypteridaceae

Salino & T.E.Almeida comb. nov.

urn:lsid:ipni.org:names:77151020-1

Thelypteris micula A.R.Sm., Fieldiana, Bot., n.s., 29: 33. 1992.

#### Amauropelta minima (A.R.Sm. & M.Kessler)

Taxon classificationPlantaePolypodialesThelypteridaceae

Salino & T.E.Almeida comb. nov.

urn:lsid:ipni.org:names:77151022-1

Thelypteris minima A.R.Sm. & M.Kessler, Brittonia 60(1): 55. 2008.

#### Amauropelta minutula (C.V.Morton)

Taxon classificationPlantaePolypodialesThelypteridaceae

Salino & T.E.Almeida comb. nov.

urn:lsid:ipni.org:names:77151024-1

Thelypteris minutula C.V.Morton, Amer. Fern J. 43(4): 173. 1953.

#### Amauropelta mombachensis (L.D.Gómez)

Taxon classificationPlantaePolypodialesThelypteridaceae

Salino & T.E.Almeida comb. nov.

urn:lsid:ipni.org:names:77151286-1

Thelypteris mombachensis L.D.Gómez, Phytologia 52(3): 155–156. 1982.

#### Amauropelta mortonii (A.R.Sm.)

Taxon classificationPlantaePolypodialesThelypteridaceae

Salino & T.E.Almeida comb. nov.

urn:lsid:ipni.org:names:77151026-1

Thelypteris mortonii A.R.Sm., Amer. Fern J. 63(3): 125. f 11. 1973.

#### Amauropelta mosenii (C.Chr.)

Taxon classificationPlantaePolypodialesThelypteridaceae

Salino & T.E.Almeida comb. nov.

urn:lsid:ipni.org:names:77151287-1

Dryopteris mosenii C.Chr., Kongel. Danske Vidensk. Selsk. Skr., Naturvidensk. Math. Afd., ser. 7, 4: 300. f 27. 1907.
Thelypteris mosenii (C.Chr.) C.F.Reed, Phytologia 17(4): 294. 1968.

#### Amauropelta muscicola (Proctor)

Taxon classificationPlantaePolypodialesThelypteridaceae

Salino & T.E.Almeida comb. nov.

urn:lsid:ipni.org:names:77151028-1

Thelypteris muscicola Proctor, Rhodora 63: 33. 1961.

#### Amauropelta namaphila (Proctor)

Taxon classificationPlantaePolypodialesThelypteridaceae

Salino & T.E.Almeida comb. nov.

urn:lsid:ipni.org:names:77151030-1

Thelypteris namaphila Proctor, Amer. Fern J. 75(2): 56. f 1. 1985.

#### Amauropelta neglecta (Brade & Rosenst.)

Taxon classificationPlantaePolypodialesThelypteridaceae

Salino & T.E.Almeida comb. nov.

urn:lsid:ipni.org:names:77151032-1

Dryopteris neglecta Brade & Rosenst., Bol. Mus. Nac. Rio de Janeiro 7(3): 142, t.1, f 3, t. 7. 1931.
Thelypteris neglecta (Brade & Rosenst.) Ching, Bull. Fan Mem. Inst. Biol. Bot. 10: 253. 1941.

#### Amauropelta negligens (Jenman)

Taxon classificationPlantaePolypodialesThelypteridaceae

Salino & T.E.Almeida comb. nov.

urn:lsid:ipni.org:names:77151033-1

Nephrodium negligens Jenman, Bull. Bot. Dept. 3: 21. 1896.
Thelypteris negligens (Jenman) Proctor, Amer. Fern J. 71(2): 58. 1981.

#### Amauropelta nephelium (A.R.Sm. & M.Kessler)

Taxon classificationPlantaePolypodialesThelypteridaceae

Salino & T.E.Almeida comb. nov.

urn:lsid:ipni.org:names:77151036-1

Thelypteris nephelium A.R.Sm. & M.Kessler, Brittonia 60(1): 57. 2008.

#### Amauropelta nitens (Desv.)

Taxon classificationPlantaePolypodialesThelypteridaceae

Salino & T.E.Almeida comb. nov.

urn:lsid:ipni.org:names:77151038-1

Polypodium nitens Desv., Mém. Soc. Linn. Paris 6: 240. 1827.
Thelypteris nitens (Desv.) R.M.Tryon, Rhodora 69(777): 7. 1967.

#### Amauropelta novaeana (Brade)

Taxon classificationPlantaePolypodialesThelypteridaceae

Salino & T.E.Almeida comb. nov.

urn:lsid:ipni.org:names:77151288-1

Dryopteris novaeana Brade, Arq. Inst. Biol. Veg. 3: 2, t. 2, 4(4-6), 6(4-5). 1936.
Thelypteris novaena (Brade) Ponce, Novon 8(3): 275. 1998.

#### Amauropelta nubicola (de la Sota)

Taxon classificationPlantaePolypodialesThelypteridaceae

Salino & T.E.Almeida comb. nov.

urn:lsid:ipni.org:names:77151040-1

Thelypteris nubicola de la Sota, Darwiniana 18: 225. pl 4, 8b. 1973.

#### Amauropelta nubigena (A.R.Sm.)

Taxon classificationPlantaePolypodialesThelypteridaceae

Salino & T.E.Almeida comb. nov.

urn:lsid:ipni.org:names:77151042-1

Thelypteris nubigena A.R.Sm., Proc. Calif. Acad. Sci., ser. 4, 40(8): 229. f 7C. 1975.

#### Amauropelta oaxacana (A.R.Sm.)

Taxon classificationPlantaePolypodialesThelypteridaceae

Salino & T.E.Almeida comb. nov.

urn:lsid:ipni.org:names:77151044-1

Thelypteris oaxacana A.R.Sm., Amer. Fern J. 63(3): 125. f 7-8. 1973.

#### Amauropelta ophiorhizoma (A.R.Sm. & Lellinger)

Taxon classificationPlantaePolypodialesThelypteridaceae

Salino & T.E.Almeida comb. nov.

urn:lsid:ipni.org:names:77151046-1

Thelypteris ophiorhizoma A.R.Sm. & Lellinger, Proc. Biol. Soc. Wash. 98(4): 925. f 8. 1985.

#### Amauropelta oviedoae (C.Sánchez & Zavaro)

Taxon classificationPlantaePolypodialesThelypteridaceae

Salino & T.E.Almeida comb. nov.

urn:lsid:ipni.org:names:77151048-1

Thelypteris oviedoae C.Sánchez & Zavaro, Fontqueria 31: 225. f 2. 1991.

#### Amauropelta pachyrhachis (Kunze ex Mett.)

Taxon classificationPlantaePolypodialesThelypteridaceae

Salino & T.E.Almeida comb. nov.

urn:lsid:ipni.org:names:77151289-1

Aspidium pachyrhachis Kunze ex Mett., Abh. Senckenberg. Naturf. Ges. 2: 367. 1858.
Thelypteris pachyrhachis (Kunze ex Mett.) Ching, Bull. Fan Mem. Inst. Biol. 10: 253. 1941.

#### Amauropelta paleacea (A.R.Sm.)

Taxon classificationPlantaePolypodialesThelypteridaceae

Salino & T.E.Almeida comb. nov.

urn:lsid:ipni.org:names:77151049-1

Thelypteris paleacea A.R.Sm., Fl. Ecuador 18: 62. 1983.

#### Amauropelta patula (Fée)

Taxon classificationPlantaePolypodialesThelypteridaceae

Salino & T.E.Almeida comb. nov.

urn:lsid:ipni.org:names:77151052-1

Gymnogramma patula Fée, Crypt. vasc. Brésil 1: 59, t. 14, f. 3. 1869.

#### Amauropelta pavoniana (Klotzsch)

Taxon classificationPlantaePolypodialesThelypteridaceae

Salino & T.E.Almeida comb. nov.

urn:lsid:ipni.org:names:77151054-1

Polypodium pavonianum Klotzsch, Linnaea 20. 386. 1847.
Thelypteris pavoniana (Klotzsch) R.M.Tryon, Rhodora 69(777): 7. 1967.

#### Amauropelta pelludia (A.R.Sm. & M.Kessler)

Taxon classificationPlantaePolypodialesThelypteridaceae

Salino & T.E.Almeida comb. nov.

urn:lsid:ipni.org:names:77151056-1

Thelypteris pelludia A.R.Sm. & M.Kessler, Brittonia 60(1): 58. 2008.

#### Amauropelta peradenia (A.R.Sm.)

Taxon classificationPlantaePolypodialesThelypteridaceae

A.R.Sm. comb. nov.

urn:lsid:ipni.org:names:77151058-1

Thelypteris peradenia A.R.Sm., Ann. Missouri Bot. Gard. 77(2): 272, f. 11. 1990.

#### Amauropelta peruviana (Rosenst.)

Taxon classificationPlantaePolypodialesThelypteridaceae

Salino & T.E.Almeida comb. nov.

urn:lsid:ipni.org:names:77151292-1

Dryopteris peruviana Rosenst., Repert. Spec. Nov. Regni Veg. 7: 298-299. 1909.
Thelypteris peruviana (Rosenst.) R.M.Tryon, Rhodora 69(777): 7. 1967.

#### Amauropelta phacelothrix (Rosenst.)

Taxon classificationPlantaePolypodialesThelypteridaceae

Salino & T.E.Almeida comb. nov.

urn:lsid:ipni.org:names:77151293-1

Dryopteris phacelothrix Rosenst., Repert. Spec. Nov. Regni Veg. 11: 56. 1912.
Thelypteris phacelothrix (Rosenst.) R.M.Tryon, Rhodora 69(777): 7. 1967.

#### Amauropelta physematioides (Kuhn & Christ ex Krug)

Taxon classificationPlantaePolypodialesThelypteridaceae

Salino & T.E.Almeida comb. nov.

urn:lsid:ipni.org:names:77151060-1

Aspidium physematioides Kuhn & Christ ex Krug, Bot. Jahrb. Syst. 24: 115. 1897.
Thelypteris physematioides (Kuhn & Christ ex Krug) C.V.Morton, Amer. Fern J. 43(4): 174. 1953.

#### Amauropelta piedrensis (C.Chr.)

Taxon classificationPlantaePolypodialesThelypteridaceae

Salino & T.E.Almeida comb. nov.

urn:lsid:ipni.org:names:77151294-1

Dryopteris piedrensis C.Chr., Smithsonian Misc. Collect. 52: 372. 1909.
Thelypteris piedrensis (C.Chr.) C.V.Morton, Amer. Fern J. 53(2): 69. 1963.

#### Amauropelta pilosissima (C.V.Morton)

Taxon classificationPlantaePolypodialesThelypteridaceae

A.R.Sm. comb. nov.

urn:lsid:ipni.org:names:77151062-1

Thelypteris pilosissima C.V.Morton, Fieldiana, Botany 28: 11. 1951.

#### Amauropelta pilosohispida (Hook.)

Taxon classificationPlantaePolypodialesThelypteridaceae

A.R.Sm. comb. nov.

urn:lsid:ipni.org:names:77151421-1

Nephrodium pilosohispidum Hook., Spec. Fil. 4: 105. 1862.
Thelypteris pilosohispida (Hook.) Alston, J. Wash. Acad. Sci. 48(7): 233. 1958.

#### Amauropelta pleiophylla (Sehnem)

Taxon classificationPlantaePolypodialesThelypteridaceae

Salino & T.E.Almeida comb. nov.

urn:lsid:ipni.org:names:77151295-1

Dryopteris pleiophylla Sehnem, Fl. Ilustr. Catarin. 1(Aspid.): 245. f 61. 1979.
Thelypteris pleiophylla (Sehnem) Ponce, Darwiniana 33(1-4): 270. 1995.

#### Amauropelta podotricha (Sehnem)

Taxon classificationPlantaePolypodialesThelypteridaceae

Salino & T.E.Almeida comb. nov.

urn:lsid:ipni.org:names:77151296-1

Dryopteris podotricha Sehnem, Fl. Ilustr. Catarin. 1(Aspid.): 232. f 57. 1979.
Thelypteris podotricha (Sehnem) Ponce, Darwiniana 33(1-4): 270. 1995.

#### Amauropelta proboscidea (A.R.Sm.)

Taxon classificationPlantaePolypodialesThelypteridaceae

Salino & T.E.Almeida comb. nov.

urn:lsid:ipni.org:names:77151064-1

Thelypteris proboscidea A.R.Sm., Fieldiana, Bot., n.s., 29: 36. 1992.

#### Amauropelta proctorii (A.R.Sm. & Lellinger)

Taxon classificationPlantaePolypodialesThelypteridaceae

A.R.Sm. comb. nov.

urn:lsid:ipni.org:names:77151297-1

Thelypteris proctorii A.R.Sm. & Lellinger, Proc. Biol. Soc. Wash. 98(4): 925. f 9. 1985.

#### Amauropelta prolatipedis (Lellinger)

Taxon classificationPlantaePolypodialesThelypteridaceae

A.R.Sm. comb. nov.

urn:lsid:ipni.org:names:77151298-1

Thelypteris prolatipedis Lellinger, Amer. Fern J. 67(2): 60, 1977.
Dryopteris coarctata var. longipes C.Chr., Kongel. Danske Vidensk. Selsk. Skr., Naturvidensk. Math. Afd., ser. 7, 4: 292. 1907.

#### Amauropelta ptarmiciformis (C.Chr. & Rosenst. ex Rosenst.)

Taxon classificationPlantaePolypodialesThelypteridaceae

Salino & T.E.Almeida comb. nov.

urn:lsid:ipni.org:names:77151065-1

Dryopteris ptarmiciformis C.Chr. & Rosenst. ex Rosenst., Repert. Spec. Nov. Regni Veg. 12: 472. 1913.
Thelypteris ptarmiciformis (C.Chr. & Rosenst. ex Rosenst.) C.F.Reed, Phytologia 17(4): 307. 1968.

#### Amauropelta pteroidea (Klotzsch)

Taxon classificationPlantaePolypodialesThelypteridaceae

A.R.Sm. comb. nov.

urn:lsid:ipni.org:names:77151068-1

Polypodium pteroideum Klotzsch, Linnaea 20. 389. 1847.
Thelypteris pteroidea (Klotzsch) R.M.Tryon, Rhodora 69(777): 8. 1967.

#### Amauropelta pusilla (Mett.)

Taxon classificationPlantaePolypodialesThelypteridaceae

A.R.Sm. comb. nov.

urn:lsid:ipni.org:names:77151070-1

Aspidium pusillum Mett., Ann. Sci. Nat., Bot., sér. 5, 2: 245. 1864.
Thelypteris pusilla (Mett.) Ching, Bull. Fan Mem. Inst. Biol. 10: 254. 1941.

#### Amauropelta raddii (Rosenst.)

Taxon classificationPlantaePolypodialesThelypteridaceae

Salino & T.E.Almeida comb. nov.

urn:lsid:ipni.org:names:77151072-1

Dryopteris raddii Rosenst., Hedwigia 56: 367. 1915.
Thelypteris raddii (Rosenst.) Ponce, Darwiniana 33(1-4): 266. 1995.

#### Amauropelta randallii (Maxon & C.V.Morton ex C.V.Morton)

Taxon classificationPlantaePolypodialesThelypteridaceae

Salino & T.E.Almeida comb. nov.

urn:lsid:ipni.org:names:77151074-1

Thelypteris randallii Maxon & C.V.Morton ex C.V.Morton, Amer. Fern J. 53(2): 69. 1963.

#### Amauropelta recumbens (Rosenst.)

Taxon classificationPlantaePolypodialesThelypteridaceae

Salino & T.E.Almeida comb. nov.

urn:lsid:ipni.org:names:77151299-1

Dryopteris recumbens Rosenst., Hedwigia 46. 123. 1906.
Thelypteris recumbens (Rosenst.) C.F.Reed, Phytologia 17: 308. 1968.

#### Amauropelta reducta (C.Chr.)

Taxon classificationPlantaePolypodialesThelypteridaceae

Salino & T.E.Almeida comb. nov.

urn:lsid:ipni.org:names:77151076-1

Dryopteris reducta C.Chr., Kongl. Svenska Vetenskapsakad. Handl., ser. 3, 16(2): 18, t. 2, f. 1-3. 1937.

#### Amauropelta regnelliana (C.Chr.)

Taxon classificationPlantaePolypodialesThelypteridaceae

Salino & T.E.Almeida comb. nov.

urn:lsid:ipni.org:names:77151300-1

Dryopteris regnelliana C.Chr., Kongel. Danske.Vidensk. Selsk. Skr., Naturvidensk. Math. Afd., ser. 7, 4: 284, f. 12. 1907.
Thelypteris regnelliana (C.Chr.) Ponce, Darwiniana 33(1-4): 264. 1995.

#### Amauropelta retrorsa (Sodiro)

Taxon classificationPlantaePolypodialesThelypteridaceae

Salino & T.E.Almeida comb. nov.

urn:lsid:ipni.org:names:77151078-1

Nephrodium retrorsum Sodiro, Recens. Crypt. Vasc. Quit. 51. 1883.
Thelypteris retrorsa (Sodiro) A.R.Sm., Fl. Ecuador 18: 71. 1983.

#### Amauropelta rheophyta (Proctor)

Taxon classificationPlantaePolypodialesThelypteridaceae

Salino & T.E.Almeida comb. nov.

urn:lsid:ipni.org:names:77151080-1

Thelypteris rheophyta Proctor, Amer. Fern J. 75(2): 58. f 2. 1985.

#### Amauropelta rigescens (Sodiro)

Taxon classificationPlantaePolypodialesThelypteridaceae

Salino & T.E.Almeida comb. nov.

urn:lsid:ipni.org:names:77151081-1

Nephrodium rigescens Sodiro, Crypt. Vasc. Quit. 239. 1893.
Thelypteris rigescens (Sodiro) A.R.Sm., Fl. Ecuador 18: 72. 1983.

#### Amauropelta rivularioides (Fée)

Taxon classificationPlantaePolypodialesThelypteridaceae

Salino & T.E.Almeida comb. nov.

urn:lsid:ipni.org:names:77151084-1

Aspidium rivularioides Fée, Crypt. Vasc. Brésil 1: 145, t. 50, f. 1. 1869.
Thelypteris rivularioides (Fée) Abbiatti, Revista Mus. La Plata, Secc. Bot. 9(36-37): 19. 1958.

#### Amauropelta roraimensis (Baker)

Taxon classificationPlantaePolypodialesThelypteridaceae

A.R.Sm. comb. nov.

urn:lsid:ipni.org:names:77151301-1

Polypodium roraimense Baker, Timehri 5: 214. 1886.
Thelypteris roraimensis (Baker) C.F.Reed, Phytologia 17(4): 310. 1968.

#### Amauropelta rosenstockii (C.Chr.)

Taxon classificationPlantaePolypodialesThelypteridaceae

Salino & T.E.Almeida comb. nov.

urn:lsid:ipni.org:names:77151086-1

Dryopteris rosenstockii C.Chr., Kongel. Danske.Vidensk. Selsk. Skr., Naturvidensk. Math. Afd., ser. 7, 4: 304, f. 30. 1907.
Thelypteris rosenstockii (C.Chr.) R.M.Tryon, Rhodora 69(777): 8. 1977.

#### Amauropelta rosulata (A.R.Sm. & M.Kessler)

Taxon classificationPlantaePolypodialesThelypteridaceae

Salino & T.E.Almeida comb. nov.

urn:lsid:ipni.org:names:77151088-1

Thelypteris rosulata A.R.Sm. & M.Kessler, Brittonia 60(1): 60. 2008.

#### Amauropelta rudiformis (C.Chr.)

Taxon classificationPlantaePolypodialesThelypteridaceae

Salino & T.E.Almeida comb. nov.

urn:lsid:ipni.org:names:77151090-1

Dryopteris rudiformis C.Chr., Univ. Calif. Publ. Bot. 19(9): 305. 1941.
Thelypteris rudiformis (C.Chr.) A.R.Sm., Fl. Ecuador 18: 74. 1983.

#### Amauropelta rufa (Poir.)

Taxon classificationPlantaePolypodialesThelypteridaceae

Salino & T.E.Almeida comb. nov.

urn:lsid:ipni.org:names:77151302-1

Polypodium rufum Poir., Encycl. 5: 532. 1804.
Thelypteris rufa (Poir.) A.R.Sm. Fl. Ecuador 18: 77. 1983.

#### Amauropelta ruiziana (Klotzsch)

Taxon classificationPlantaePolypodialesThelypteridaceae

Salino & T.E.Almeida comb. nov.

urn:lsid:ipni.org:names:77151092-1

Polypodium ruizianum Klotzsch, Linnaea 20: 385. 1847.
Thelypteris ruiziana (Klotzsch) A.R.Sm. Fieldiana, Bot., n.s. 29: 35. 1992.

#### Amauropelta rupestris (Klotzsch)

Taxon classificationPlantaePolypodialesThelypteridaceae

A.R.Sm. comb. nov.

urn:lsid:ipni.org:names:77151094-1

Leptogramma rupestris Klotzsch, Linnaea 20. 415. 1847.
Thelypteris rupestris (Klotzsch) C.F.Reed, Phytologia 17(4): 310. 1968.

#### Amauropelta rupicola (C.Chr.)

Taxon classificationPlantaePolypodialesThelypteridaceae

Salino & T.E.Almeida comb. nov.

urn:lsid:ipni.org:names:77151303-1

Dryopteris rupicola C.Chr., Repert. Spec. Nov. Regni Veg. 15: 24. 1917.
Thelypteris rupicola (C.Chr.) Ching, Bull. Fan Mem. Inst. Biol. 10: 254. 1941.

#### Amauropelta rustica (Fée)

Taxon classificationPlantaePolypodialesThelypteridaceae

Salino & T.E.Almeida comb. nov.

urn:lsid:ipni.org:names:77151096-1

Phegopteris rustica Fée, Mém. Foug. 11 (Hist. Foug. Ant.): 55, t. 13, f. 1. 1866.
Thelypteris rustica (Fée) Proctor, Rhodora 61(732): 306. 1959[1960].

#### Amauropelta sanctae-catharinae (Rosenst.)

Taxon classificationPlantaePolypodialesThelypteridaceae

Salino & T.E.Almeida comb. nov.

urn:lsid:ipni.org:names:77151097-1

Dryopteris sanctae-catharinae Rosenst., Hedwigia 46: 126. 1906.
Thelypteris sanctae-catharinae (Rosenst.) Ponce, Darwiniana 33(1-4): 280. 1995.

#### Amauropelta saxicola (Sw.)

Taxon classificationPlantaePolypodialesThelypteridaceae

Salino & T.E.Almeida comb. nov.

urn:lsid:ipni.org:names:77151100-1

Polypodium saxicola Sw., Kongl. Vetensk. Acad. Handl. 1817: 59, t. 3, f. 5. 1817.
Thelpyteris saxicola (Sw.) C.F.Reed, Phytologia 17(4): 312. 1968.

#### Amauropelta scalpturoides (Fée)

Taxon classificationPlantaePolypodialesThelypteridaceae

Salino & T.E.Almeida comb. nov.

urn:lsid:ipni.org:names:77151102-1

Phegopteris scalpturoides Fée, Mém. foug. 11 (Hist. foug. Ant.): 51. 1866.
Thelypteris scalpturoides (Fée) C.F.Reed, Phytologia 17(4): 312. 1968.

#### Amauropelta sellensis (C.Chr.)

Taxon classificationPlantaePolypodialesThelypteridaceae

Salino & T.E.Almeida comb. nov.

urn:lsid:ipni.org:names:77151104-1

Dryopteris sellensis C.Chr., Kungl. Svenska Vetenskapsakad. Handl., ser. 3, 16(2): 24. t. 3(7−8). 1937.

#### Amauropelta semilunata (Sodiro)

Taxon classificationPlantaePolypodialesThelypteridaceae

Salino & T.E.Almeida comb. nov.

urn:lsid:ipni.org:names:77151106-1

Nephrodium semilunatum Sodiro, Recens. Crypt. Vasc. Quit. 46. 1883.
Thelyteris semilunata (Sodiro) A.R.Sm., Fl. Ecuador 18: 79. 1983.

#### Amauropelta shaferi (Maxon & C.Chr.)

Taxon classificationPlantaePolypodialesThelypteridaceae

Salino & T.E.Almeida comb. nov.

urn:lsid:ipni.org:names:77151304-1

Dryopteris shaferi Maxon & C.Chr., Amer. Fern J. 4: 77. 1914.
Thelypteris shaferi (Maxon & C.Chr.) Duek, Adansonia, sér. 2, 11: 719. 1971 [1972].

#### Amauropelta soridepressa (Salino & V.A.O.Dittrich)

Taxon classificationPlantaePolypodialesThelypteridaceae

Salino & T.E.Almeida comb. nov.

urn:lsid:ipni.org:names:77151108-1

Thelypteris soridepressa Salino & V.A.O.Dittrich, Amer. Fern J. 98(4): 199. f 1. 2009.

#### Amauropelta steyermarkii (A.R.Sm.)

Taxon classificationPlantaePolypodialesThelypteridaceae

Salino & T.E.Almeida comb. nov.

urn:lsid:ipni.org:names:77151110-1

Thelypteris steyermarkii A.R.Sm., Fl. Ecuador 18: 79. 1983.

#### Amauropelta stierii (Rosenst.)

Taxon classificationPlantaePolypodialesThelypteridaceae

Salino & T.E.Almeida comb. nov.

urn:lsid:ipni.org:names:77151305-1

Gymnogramma stierii Rosenst., Festschrift Albert von Bamberg 64. 1905.
Thelypteris stierii (Rosenst.) C.F.Reed, Phytologia 17(4): 316. 1968.

#### Amauropelta straminea (Sodiro)

Taxon classificationPlantaePolypodialesThelypteridaceae

Salino & T.E.Almeida comb. nov.

urn:lsid:ipni.org:names:77151112-1

Nephrodium stramineum Sodiro, Recens. Crypt. Vasc. Quit. 43. 1883.
Thelypteris chimboracensis A.R.Sm., Fl. Ecuador 18: 34. 1993.

#### Amauropelta strigillosa (A.R.Sm. & Lellinger)

Taxon classificationPlantaePolypodialesThelypteridaceae

Salino & T.E.Almeida comb. nov.

urn:lsid:ipni.org:names:77151113-1

Thelypteris strigillosa A.R.Sm. & Lellinger, Proc. Biol. Soc. Wash. 98(4): 927. F 10. 1985.

#### Amauropelta struthiopteroides (C.Chr.)

Taxon classificationPlantaePolypodialesThelypteridaceae

Salino & T.E.Almeida comb. nov.

urn:lsid:ipni.org:names:77151306-1

Dryopteris struthiopteroides C.Chr., Smithsonian Misc. Collect. 52(3): 388. 1909.
Thelypteris struthiopteroides (C.Chr.) C.F.Reed, Phytologia 17(4): 316. 1968.

#### Amauropelta subscandens (A.R.Sm.)

Taxon classificationPlantaePolypodialesThelypteridaceae

Salino & T.E.Almeida comb. nov.

urn:lsid:ipni.org:names:77151116-1

Thelypteris subscandens A.R.Sm., Ann. Missouri Bot. Gard. 77(1): 123. f 4. 1990.

#### Amauropelta subtilis (A.R.Sm.)

Taxon classificationPlantaePolypodialesThelypteridaceae

Salino & T.E.Almeida comb. nov.

urn:lsid:ipni.org:names:77151118-1

Thelypteris subtilis A.R.Sm., Fl. Ecuador 18: 80. 1983.

#### Amauropelta supina (Sodiro)

Taxon classificationPlantaePolypodialesThelypteridaceae

Salino & T.E.Almeida comb. nov.

urn:lsid:ipni.org:names:77151307-1

Nephrodium supinum Sodiro, Crypt. Vasc. Quit. 241. 1893.
Thelypteris supina (Sodiro) A.R.Sm., Fl. Ecuador 18: 82. 1983.

#### Amauropelta tablana (Christ)

Taxon classificationPlantaePolypodialesThelypteridaceae

Salino & T.E.Almeida comb. nov.

urn:lsid:ipni.org:names:77151120-1

Aspidium tablanum Christ, Bull. Herb. Boissier, sér. 2, 5: 727. 1905.
Thelypteris tablana (Christ) A.R.Sm., Amer. Fern J. 63(3): 127. 1973.

#### Amauropelta tamandarei (Rosenst.)

Taxon classificationPlantaePolypodialesThelypteridaceae

Salino & T.E.Almeida comb. nov.

urn:lsid:ipni.org:names:77151122-1

Dryopteris tamandarei Rosenst., Hedwigia 56. 365. 1915.
Thelypteris tamandarei (Rosenst.) Ponce, Novon 8(3): 277. 1998.

#### Amauropelta tapantensis (A.R.Sm. & Lellinger)

Taxon classificationPlantaePolypodialesThelypteridaceae

Salino & T.E.Almeida comb. nov.

urn:lsid:ipni.org:names:77151124-1

Thelypteris tapantensis A.R.Sm. & Lellinger, Proc. Biol. Soc. Wash. 98(4): f 11. 927. 1985.

#### Amauropelta tenerrima (Fée)

Taxon classificationPlantaePolypodialesThelypteridaceae

Salino & T.E.Almeida comb. nov.

urn:lsid:ipni.org:names:77151126-1

Aspidium tenerrimum Fée, Crypt. Vasc. Brésil 1: 134. t 43, f 1. 1869.
Thelypteris tenerrima (Fée) C.F.Reed, Phytologia 17(4): 319. 1968.

#### Amauropelta trelawniensis (Proctor)

Taxon classificationPlantaePolypodialesThelypteridaceae

Salino & T.E.Almeida comb. nov.

urn:lsid:ipni.org:names:77151308-1

Thelypteris trelawniensis Proctor, Amer. Fern J. 71(2): 58. 1981.

#### Amauropelta uncinata (A.R.Sm.)

Taxon classificationPlantaePolypodialesThelypteridaceae

Salino & T.E.Almeida comb. nov.

urn:lsid:ipni.org:names:77151128-1

Thelypteris uncinata A.R.Sm., Fl. Ecuador 18: 86. 1983.

#### Amauropelta vattuonei (Hicken)

Taxon classificationPlantaePolypodialesThelypteridaceae

Salino & T.E.Almeida comb. nov.

urn:lsid:ipni.org:names:77151129-1

Dryopteris vattuonei Hicken, Darwiniana 1: 100. 1924.
Thelypteris vattuonei (Hicken) Abbiatti, Darwiniana 13: 566. 1964.

#### Amauropelta venturae (A.R.Sm.)

Taxon classificationPlantaePolypodialesThelypteridaceae

Salino & T.E.Almeida comb. nov.

urn:lsid:ipni.org:names:77151132-1

Thelypteris venturae A.R.Sm., Mem. New York Bot. Gard. 88: 638. f 302A-C. 2004.

#### Amauropelta vernicosa (A.R.Sm. & Lellinger)

Taxon classificationPlantaePolypodialesThelypteridaceae

Salino & T.E.Almeida comb. nov.

urn:lsid:ipni.org:names:77151134-1

Thelypteris vernicosa A.R.Sm. & Lellinger, Proc. Biol. Soc. Wash. 98(4): 929. f 12. 1985.

#### Amauropelta villana (L.D.Gómez)

Taxon classificationPlantaePolypodialesThelypteridaceae

Salino & T.E.Almeida comb. nov.

urn:lsid:ipni.org:names:77151136-1

Thelypteris villana L.D.Gómez, Rev. Biol. Trop. 17(1): 106. f 1-4. 1970.

#### Amauropelta yungensis (A.R.Sm. & M.Kessler)

Taxon classificationPlantaePolypodialesThelypteridaceae

Salino & T.E.Almeida comb. nov.

urn:lsid:ipni.org:names:77151138-1

Thelypteris yungensis A.R.Sm. & M.Kessler, Brittonia 60(1): 61. 2008.

#### Amauropelta zurquiana (A.R.Sm. & Lellinger)

Taxon classificationPlantaePolypodialesThelypteridaceae

Salino & T.E.Almeida comb. nov.

urn:lsid:ipni.org:names:77151140-1

Thelypteris zurquiana A.R.Sm. & Lellinger, Proc. Biol. Soc. Wash. 98(4): 929. f 13. 1985.

### New combinations in Goniopteris

#### Goniopteris abdita (Proctor)

Taxon classificationPlantaePolypodialesThelypteridaceae

Salino & T.E.Almeida comb. nov.

urn:lsid:ipni.org:names:77151141-1

Thelypteris abdita Proctor Amer. Fern J. 75(2): 63. f 4. 1985.

#### Goniopteris abrupta (Desv.)

Taxon classificationPlantaePolypodialesThelypteridaceae

A.R.Sm. comb. nov.

urn:lsid:ipni.org:names:77151144-1

Polypodium abruptum Desv., Mém. Soc. Linn. Paris 6: 239. 1827.
Thelypteris abrupta (Desv.) Proctor, Rhodora 61: 305. 1960.

#### Goniopteris alan-smithiana (L.D.Gómez)

Taxon classificationPlantaePolypodialesThelypteridaceae

Salino & T.E.Almeida comb. nov.

urn:lsid:ipni.org:names:77151354-1

Thelypteris alan-smithiana L.D.Gómez, Phytologia 50(7): 458. 1982.

#### Goniopteris amazonica (Salino & R.S.Fernandes)

Taxon classificationPlantaePolypodialesThelypteridaceae

Salino & T.E.Almeida comb. nov.

urn:lsid:ipni.org:names:77151310-1

Thelypteris amazonica Salino & R.S.Fernandes, Nordic J. Bot. 29(5): 611. f 1-2. 2011.

#### Goniopteris ancyriothrix (Rosenst.)

Taxon classificationPlantaePolypodialesThelypteridaceae

Salino & T.E.Almeida comb. nov.

urn:lsid:ipni.org:names:77151311-1

Dryopteris ancyriothrix Rosenst., Repert. Spec. Nov. Regni Veg. 7: 305-306. 1909.
Thelypteris ancyriothrix (Rosenst.) A.R.Sm., Fl. Ecuador 18: 140. 1983.

#### Goniopteris anoptera (Kunze ex Kuhn)

Taxon classificationPlantaePolypodialesThelypteridaceae

Salino & T.E.Almeida comb. nov.

urn:lsid:ipni.org:names:77151312-1

Aspidium anopteron Kunze ex Kuhn, Linnaea 36. 113. 1869.
Thelypteris anoptera (Kunze ex Kuhn) C.F.Reed, Phytologia 17(4): 260. 1968.

#### Goniopteris aureola (A.R.Sm.)

Taxon classificationPlantaePolypodialesThelypteridaceae

Salino & T.E.Almeida comb. nov.

urn:lsid:ipni.org:names:77151148-1

Thelypteris aureola A.R.Sm., Ann. Missouri Bot. Gard. 77(1): 118. f 1D-E. 1990.

#### Goniopteris beckeriana (F.B.Matos, A.R.Sm. & Labiak)

Taxon classificationPlantaePolypodialesThelypteridaceae

Salino & T.E.Almeida comb. nov.

urn:lsid:ipni.org:names:77151150-1

Thelypteris beckeriana F.B.Matos, A.R.Sm. & Labiak, Brittonia 62(2): 149. f 1. 2010.

#### Goniopteris berlinii (A.R.Sm.)

Taxon classificationPlantaePolypodialesThelypteridaceae

Salino & T.E.Almeida comb. nov.

urn:lsid:ipni.org:names:77151152-1

Thelypteris berlinii A.R.Sm., Novon 16: 431. 2006.

#### Goniopteris bibrachiata (Jenman)

Taxon classificationPlantaePolypodialesThelypteridaceae

Salino & T.E.Almeida comb. nov.

urn:lsid:ipni.org:names:77151154-1

Nephrodium bibrachiatum Jenman, Gard. Chron., ser. 3, 15: 230. 1894.
Thelypteris bibrachiata (Jenman) Proctor, Ferns Jamaica 337. 1985.

#### Goniopteris biformata (Rosenst.)

Taxon classificationPlantaePolypodialesThelypteridaceae

Salino & T.E.Almeida comb. nov.

urn:lsid:ipni.org:names:77151355-1

Dryopteris biformata Rosenst., Repert. Spec. Nov. Regni Veg. 7: 300-301. 1909.
Thelypteris biformata (Rosenst.) R.M.Tryon, Rhodora 69(777): 5. 1967.

#### Goniopteris blanda (Fée)

Taxon classificationPlantaePolypodialesThelypteridaceae

Salino & T.E.Almeida comb. nov.

urn:lsid:ipni.org:names:77151156-1

Phegopteris blanda Fée, Mém. Foug. 8: 91. 1857.
Thelypteris blanda (Fée) C.F.Reed, Phytologia 17(4): 264. 1968.

#### Goniopteris calypso (L.D.Gómez)

Taxon classificationPlantaePolypodialesThelypteridaceae

Salino & T.E.Almeida comb. nov.

urn:lsid:ipni.org:names:77151157-1

Thelypteris calypso L.D.Gómez, Brenesia 8: 98. 1976.

#### Goniopteris chocoensis (A.R.Sm. & Lellinger)

Taxon classificationPlantaePolypodialesThelypteridaceae

Salino & T.E.Almeida comb. nov.

urn:lsid:ipni.org:names:77151160-1

Thelypteris chocoensis A.R.Sm. & Lellinger, Proc. Biol. Soc. Wash. 98(4): 916. f 1. 1985.

#### Goniopteris clypeata (Maxon & C.V.Morton)

Taxon classificationPlantaePolypodialesThelypteridaceae

Salino & T.E.Almeida comb. nov.

urn:lsid:ipni.org:names:77151162-1

Dryopteris clypeata Maxon & C.V.Morton, Bull. Torrey Bot. Club 66: 52. 1939.
Thelypteris clypeata (Maxon & C.V.Morton) K.U.Kramer, Acta Bot. Neerl. 18: 141. 1969.

#### Goniopteris cordata (Fée)

Taxon classificationPlantaePolypodialesThelypteridaceae

Salino & T.E.Almeida comb. nov.

urn:lsid:ipni.org:names:77151164-1

Phegopteris cordata Fée, Mém. Foug. 5 (Gen. filic.): 244. 1852.
Thelypteris cordata (Fée) Proctor, Bull. Inst. Jamaica, Sci. Ser. 5: 59. 1953.

#### Goniopteris costaricensis

Taxon classificationPlantaePolypodialesThelypteridaceae

Salino & T.E.Almeida nom. nov.

urn:lsid:ipni.org:names:77151356-1

Thelypteris crenata A.R.Sm. & Lellinger, Proc. Biol. Soc. Wash. 98(4): 919. f 4. 1985.
Goniopteris costaricensis This needs a new name in Goniopteris because of the prior Goniopteris crenata C. Presl, 1836.

#### Goniopteris crassipila (Caluff & C.Sánchez)

Taxon classificationPlantaePolypodialesThelypteridaceae

Salino & T.E.Almeida comb. nov.

urn:lsid:ipni.org:names:77151314-1

Thelypteris crassipila Caluff & C.Sánchez, Willdenowia 34: 513. 2004.

#### Goniopteris croatii (A.R.Sm.)

Taxon classificationPlantaePolypodialesThelypteridaceae

Salino & T.E.Almeida comb. nov.

urn:lsid:ipni.org:names:77151168-1

Thelypteris croatii A.R.Sm., Ann. Missouri Bot. Gard. 77(1): 122. f 1A-C. 1990.

#### Goniopteris curta (Christ)

Taxon classificationPlantaePolypodialesThelypteridaceae

A.R.Sm. comb. nov.

urn:lsid:ipni.org:names:77151170-1

Dryopteris curta Christ, Bull. Herb. Boissier, sér. 2, 7(4): 263. 1907.
Thelypteris curta (Christ) C.F.Reed, Phytologia 17(4): 270. 1968.

#### Goniopteris dissimulans (Maxon & C.Chr.)

Taxon classificationPlantaePolypodialesThelypteridaceae

Salino & T.E.Almeida comb. nov.

urn:lsid:ipni.org:names:77151172-1

Dryopteris dissimulans Maxon & C.Chr., Kongel. Danske Vidensk. Selsk. Skr., Naturvidensk. Math. Afd., ser. 7, 10: 215. 1913.
Thelypteris dissimulans (Maxon & C.Chr.) C.F.Reed, Phytologia 17(4): 273. 1968.

#### Goniopteris equitans (Christ)

Taxon classificationPlantaePolypodialesThelypteridaceae

Salino & T.E.Almeida comb. nov.

urn:lsid:ipni.org:names:77151173-1

Nephrodium equitans Christ, Bull. Herb. Boissier, sér. 2, 6(2): 163. 1906.
Thelypteris equitans (Christ) C.F.Reed, Phytologia 17(4): 275. 1968.

#### Goniopteris erythrothrix (A.R.Sm.)

Taxon classificationPlantaePolypodialesThelypteridaceae

Salino & T.E.Almeida comb. nov.

urn:lsid:ipni.org:names:77151176-1

Thelypteris erythrothrix A.R.Sm., Fieldiana, Bot., n.s., 29: 59. 1992.

#### Goniopteris gonophora (Weath.)

Taxon classificationPlantaePolypodialesThelypteridaceae

Salino & T.E.Almeida comb. nov.

urn:lsid:ipni.org:names:77151357-1

Dryopteris gonophora Weath., Lloydia 2: 164. f. 1(1−3). 1939.
Thelypteris gonophora (Weath.) A.R.Sm., Ann. Missouri Bot. Gard. 77(1): 203. 1990.

#### Goniopteris hildae (Proctor)

Taxon classificationPlantaePolypodialesThelypteridaceae

Salino & T.E.Almeida comb. nov.

urn:lsid:ipni.org:names:77151315-1

Thelypteris hildae Proctor, Amer. Fern J. 75(2): 67. f 6. 1985.

#### Goniopteris holodictya (K.U.Kramer)

Taxon classificationPlantaePolypodialesThelypteridaceae

Salino & T.E.Almeida comb. nov.

urn:lsid:ipni.org:names:77151316-1

Thelypteris holodictya K.U.Kramer, Acta Bot. Neerl. 18: 140. f. 2. 1969.

#### Goniopteris hondurensis (L.D.Gómez)

Taxon classificationPlantaePolypodialesThelypteridaceae

Salino & T.E.Almeida comb. nov.

urn:lsid:ipni.org:names:77151358-1

Thelypteris hondurensis L.D.Gómez, Phytologia 50(7): 458. 1982.

#### Goniopteris imitata (C.Chr.)

Taxon classificationPlantaePolypodialesThelypteridaceae

Salino & T.E.Almeida comb. nov.

urn:lsid:ipni.org:names:77151317-1

Dryopteris imitata C.Chr., Kongl. Svenska Vetenskapsakad. Handl., ser. 3, 16(2): 29, t. 6, f. 1-4. 1937.
Thelypteris imitata (C.Chr.) Alain, Moscosoa 3: 46. 1978.

#### Goniopteris indusiata (Salino)

Taxon classificationPlantaePolypodialesThelypteridaceae

Salino & T.E.Almeida comb. nov.

urn:lsid:ipni.org:names:77151182-1

Thelypteris indusiata Salino, Phytotaxa 156(5): 280. f 1-3. 2014.

#### Goniopteris jamesonii (Hook.)

Taxon classificationPlantaePolypodialesThelypteridaceae

Salino & T.E.Almeida comb. nov.

urn:lsid:ipni.org:names:77151184-1

Nephrodium jamesonii Hook., Sp. fil. 4: 66. 1862.
Thelypteris jamesonii (Hook.) R.M.Tryon, Rhodora 69(777): 6. 1967.

#### Goniopteris jarucoensis (Caluff & C.Sánchez)

Taxon classificationPlantaePolypodialesThelypteridaceae

Salino & T.E.Almeida comb. nov.

urn:lsid:ipni.org:names:77151186-1

Thelypteris jarucoensis Caluff & C.Sánchez, Willdenowia 34: 515. 2004.

#### Goniopteris killipii (A.R.Sm. & Lellinger)

Taxon classificationPlantaePolypodialesThelypteridaceae

Salino & T.E.Almeida comb. nov.

urn:lsid:ipni.org:names:77151188-1

Thelypteris killipii A.R.Sm. & Lellinger, Proc. Biol. Soc. Wash. 98(4): 923. f 7. 1985.

#### Goniopteris leonina (Caluff & C.Sánchez)

Taxon classificationPlantaePolypodialesThelypteridaceae

Salino & T.E.Almeida comb. nov.

urn:lsid:ipni.org:names:77151189-1

Thelypteris leonina Caluff & C.Sánchez, Willdenowia 34: 517. 2004.

#### Goniopteris levyi (E.Fourn.)

Taxon classificationPlantaePolypodialesThelypteridaceae

Salino & T.E.Almeida comb. nov.

urn:lsid:ipni.org:names:77151359-1

Aspidium levyi E.Fourn., Bull. Soc. Bot. France 19: 255. 1872.
Thelypteris levyi (E.Fourn.) C.V.Morton, Contr. U.S. Natl. Herb. 38: 37. 1967.

#### Goniopteris liebmannii (Maxon & C.V.Morton)

Taxon classificationPlantaePolypodialesThelypteridaceae

Salino & T.E.Almeida comb. nov.

urn:lsid:ipni.org:names:77151192-1

Polypodium meniscioides Liebm., Kongel. Danske Vidensk. Selsk. Skr., Naturvidensk. Math. Afd., ser. 5, 1: 211. 1849.
Dryopteris liebmannii Maxon & C.V.Morton, Bull. Torrey Bot. Club 65: 348. 1938.
Thelypteris meniscioides (Liebm.) C.F.Reed, Phytologia 17: 292. 1968.
Goniopteris liebmannii (Maxon & C.V.Morton) The earliest species ephitet, meniscioides, has been used already in Goniopteris – Goniopteris meniscioides Fée, a synonym of Ampelopteris prolifera (Retz.) Copel., a Paleotropical species.

#### Goniopteris littoralis (Salino)

Taxon classificationPlantaePolypodialesThelypteridaceae

Salino & T.E.Almeida comb. nov.

urn:lsid:ipni.org:names:77151360-1

Thelypteris littoralis Salino, Brittonia 54(4): 332. f 1. 2002.

#### Goniopteris lugubriformis (Rosenst.)

Taxon classificationPlantaePolypodialesThelypteridaceae

Salino & T.E.Almeida comb. nov.

urn:lsid:ipni.org:names:77151319-1

Dryopteris lugubriformis Rosenst., Repert. Spec. Nov. Regni Veg. 7: 299. 1909.
Thelypteris lugubriformis (Rosenst.) R.M.Tryon, Rhodora 69(777): 7. 1967.

#### Goniopteris martinezii (A.R.Sm.)

Taxon classificationPlantaePolypodialesThelypteridaceae

Salino & T.E.Almeida comb. nov.

urn:lsid:ipni.org:names:77151194-1

Thelypteris martinezii A.R.Sm., Mem. New York Bot. Gard. 88: 654-655. f 308I. 2004.

#### Goniopteris minor (C.Chr.)

Taxon classificationPlantaePolypodialesThelypteridaceae

Salino & T.E.Almeida comb. nov.

urn:lsid:ipni.org:names:77151196-1

Dryopteris nicaraguensis (E.Fourn.) C.Chr. var. minor C.Chr., Kongel. Danske Vidensk. Selsk. Skr., Naturvidensk. Math. Afd., ser. 7, 10: 252. 1913.
Thelypteris minor (C.Chr.) A.R.Sm., Phytologia 34(4): 232. 1976.

#### Goniopteris minutissima (Caluff & C.Sánchez)

Taxon classificationPlantaePolypodialesThelypteridaceae

Salino & T.E.Almeida comb. nov.

urn:lsid:ipni.org:names:77151198-1

Thelypteris minutissima Caluff & C.Sánchez, Willdenowia 34: 511. 2004.

#### Goniopteris montana (Salino)

Taxon classificationPlantaePolypodialesThelypteridaceae

Salino & T.E.Almeida comb. nov.

urn:lsid:ipni.org:names:77151361-1

Thelypteris montana Salino, Brittonia 54(4): 334. f 2D-F. 2002.

#### Goniopteris multigemmifera (Salino)

Taxon classificationPlantaePolypodialesThelypteridaceae

Salino & T.E.Almeida comb. nov.

urn:lsid:ipni.org:names:77151321-1

Thelypteris multigemmifera Salino, Brittonia 54(4): 336. f 3. 2002.

#### Goniopteris munchii (A.R.Sm.)

Taxon classificationPlantaePolypodialesThelypteridaceae

Salino & T.E.Almeida comb. nov.

urn:lsid:ipni.org:names:77151200-1

Thelypteris munchii A.R.Sm., Amer. Fern J. 63(3): 120. f 9-10. 1973.

#### Goniopteris nicaraguensis (E.Fourn.)

Taxon classificationPlantaePolypodialesThelypteridaceae

Salino & T.E.Almeida comb. nov.

urn:lsid:ipni.org:names:77151202-1

Phegopteris nicaraguensis E.Fourn., Bull. Soc. Bot. France 19: 252. 1872.
Thelypteris nicaraguensis (E.Fourn.) C.V.Morton, Contr. U.S. Natl. Herb. 38(2): 55. 1967.

#### Goniopteris nigricans (Ekman & C.Chr.)

Taxon classificationPlantaePolypodialesThelypteridaceae

Salino & T.E.Almeida comb. nov.

urn:lsid:ipni.org:names:77151204-1

Dryopteris nigricans Ekman & C.Chr., Kongl. Svenska Vetenskapsakad. Handl., ser. 3, 16(2): 31, t. 7, f. 1-2. 1937.

#### Goniopteris oroniensis (L.D.Gómez)

Taxon classificationPlantaePolypodialesThelypteridaceae

Salino & T.E.Almeida comb. nov.

urn:lsid:ipni.org:names:77151325-1

Thelypteris oroniensis L.D.Gómez, Amer. Fern J. 68(1): 9. f 1. 1978.

#### Goniopteris paranaensis (Salino)

Taxon classificationPlantaePolypodialesThelypteridaceae

Salino & T.E.Almeida comb. nov.

urn:lsid:ipni.org:names:77151323-1

Thelypteris paranaensis Salino, Brittonia 54(4): 337. F 2A-C. 2002.

#### Goniopteris paucijuga (Klotzsch)

Taxon classificationPlantaePolypodialesThelypteridaceae

A.R.Sm. comb. nov.

urn:lsid:ipni.org:names:77151205-1

Aspidium paucijugum Klotzsch, Linnaea 20: 368. 1847.
Thelypteris paucijuga (Klotzsch) A.R.Sm., Univ. Calif. Publ. Bot. 59: 98. 1971

#### Goniopteris paucipinnata (Donn.Sm.)

Taxon classificationPlantaePolypodialesThelypteridaceae

Salino & T.E.Almeida comb. nov.

urn:lsid:ipni.org:names:77151362-1

Nephrodium fendleri D.C.Eaton var. paucipinnatum Donn.Sm., Bot. Gaz. 12: 134. 1887.
Thelypteris paucipinnata (Donn.Sm.) C.F.Reed, Phytologia 17(4): 302. 1968.

#### Goniopteris pellita (Willd.)

Taxon classificationPlantaePolypodialesThelypteridaceae

A.R.Sm. comb. nov.

urn:lsid:ipni.org:names:77151324-1

Aspidium pellitum Willd., Sp. Pl. ed. 4. 5: 242. 1810.
Thelypteris pellita (Willd.) Proctor & Lourteig, Bradea 5(40): 384. 1990.

#### Goniopteris peripae (Sodiro)

Taxon classificationPlantaePolypodialesThelypteridaceae

Salino & T.E.Almeida comb. nov.

urn:lsid:ipni.org:names:77151326-1

Nephrodium peripae Sodiro, Recens. Crypt. Vasc. Quit. 52. 1883.
Thelypteris peripae (Sodiro) C.F.Reed, Phytologia 17(4): 303. 1968.

#### Goniopteris pilonensis (A.R.Sm. & M.Kessler)

Taxon classificationPlantaePolypodialesThelypteridaceae

Salino & T.E.Almeida comb. nov.

urn:lsid:ipni.org:names:77151210-1

Thelypteris pilonensis A.R.Sm. & M.Kessler, Brittonia 60(1): 58. 2008.

#### Goniopteris pinnatifida (A.R.Sm.)

Taxon classificationPlantaePolypodialesThelypteridaceae

Salino & T.E.Almeida comb. nov.

urn:lsid:ipni.org:names:77151212-1

Thelypteris pinnatifida A.R.Sm., Fl. Ecuador 18: 110. 1983.

#### Goniopteris praetermissa (Maxon)

Taxon classificationPlantaePolypodialesThelypteridaceae

Salino & T.E.Almeida comb. nov.

urn:lsid:ipni.org:names:77151214-1

Dryopteris praetermissa Maxon, Proc. Biol. Soc. Wash. 57: 20. 1944.
Thelypteris praetermissa (Maxon) A.R.Sm., Phytologia 34(3): 232. 1976.

#### Goniopteris redunca (A.R.Sm.)

Taxon classificationPlantaePolypodialesThelypteridaceae

Salino & T.E.Almeida comb. nov.

urn:lsid:ipni.org:names:77151216-1

Thelypteris redunca A.R.Sm., Ann. Missouri Bot. Gard. 77(1): 123. f 1F-H. 1990.

#### Goniopteris resiliens (Maxon)

Taxon classificationPlantaePolypodialesThelypteridaceae

Salino & T.E.Almeida comb. nov.

urn:lsid:ipni.org:names:77151218-1

Dryopteris resiliens Maxon, Field Mus. Publ. Bot. 17: 302. 1938.
Thelypteris resiliens (Maxon) A.R.Sm., Amer. Fern J. 63(3): 121. 1973.

#### Goniopteris retroflexa (L.)

Taxon classificationPlantaePolypodialesThelypteridaceae

Salino & T.E.Almeida comb. nov.

urn:lsid:ipni.org:names:77151327-1

Polypodium retroflexum L., Sp. Pl. 1089. 1753.
Thelypteris retroflexa (L.) Proctor & Lourteig, Bradea 5(40): 385. 1990.

#### Goniopteris rhachiflexuosa (Riba)

Taxon classificationPlantaePolypodialesThelypteridaceae

Salino & T.E.Almeida comb. nov.

urn:lsid:ipni.org:names:77151329-1

Thelypteris rhachiflexuosa Riba, Amer. Fern J. 79(3): 122. f 1. 1989.

#### Goniopteris ×rolandii (C.Chr.)

Taxon classificationPlantaePolypodialesThelypteridaceae

A.R.Sm. comb. nov.

urn:lsid:ipni.org:names:77151328-1

Dryopteris ×rolandii C.Chr., Kongel. Danske Vidensk. Selsk. Skr., Naturvidensk. Math. Afd., ser. 7, 10: 258. 1913.
Thelypteris ×rolandii (C.Chr.) R.M.Tryon, Rhodora 69: 8. 1967.

#### Goniopteris sapechoana (A.R.Sm. & M.Kessler)

Taxon classificationPlantaePolypodialesThelypteridaceae

Salino & T.E.Almeida comb. nov.

urn:lsid:ipni.org:names:77151220-1

Thelypteris sapechoana A.R.Sm. & M.Kessler, Brittonia 60(1): 60. 2008.

#### Goniopteris schaffneri (Fée)

Taxon classificationPlantaePolypodialesThelypteridaceae

Salino & T.E.Almeida comb. nov.

urn:lsid:ipni.org:names:77151330-1

Nephrodium schaffneri Fée, Mém. Foug. 8: 108. 1857.
Thelypteris schaffneri (Fée) C.F.Reed, Phytologia 17(4): 312. 1968.

#### Goniopteris schippii (Weath.)

Taxon classificationPlantaePolypodialesThelypteridaceae

Salino & T.E.Almeida comb. nov.

urn:lsid:ipni.org:names:77151331-1

Dryopteris schippii Weath., Amer. Fern J. 25: 52. 1935.
Thelypteris schippii (Weath.) A.R.Sm., Phytologia 34(3): 233. 1976.

#### Goniopteris schomburgkii (A.R.Sm.)

Taxon classificationPlantaePolypodialesThelypteridaceae

Salino & T.E.Almeida comb. nov.

urn:lsid:ipni.org:names:77151221-1

Thelypteris schomburgkii A.R.Sm., Ann. Missouri Bot. Gard. 77(1): 202. f 1. 1990.

#### Goniopteris schunkei (A.R.Sm.)

Taxon classificationPlantaePolypodialesThelypteridaceae

Salino & T.E.Almeida comb. nov.

urn:lsid:ipni.org:names:77151224-1

Thelypteris schunkei A.R.Sm., Fieldiana, Bot., n.s., 29: 63. 1992.

#### Goniopteris semihastata (Kunze)

Taxon classificationPlantaePolypodialesThelypteridaceae

Salino & T.E.Almeida comb. nov.

urn:lsid:ipni.org:names:77151226-1

Aspidium semihastatum Kunze, Linnaea 9: 91. 1834.
Thelypteris semihastata (Kunze) Ching, Bull. Fan Mem. Inst. Biol. 10: 254. 1941.

#### Goniopteris semirii (Salino & L.C.N.Melo)

Taxon classificationPlantaePolypodialesThelypteridaceae

Salino & T.E.Almeida comb. nov.

urn:lsid:ipni.org:names:77151332-1

Thelypteris semirii Salino & L.C.N.Melo, Novon 10(1): 74. f 1A-e. 2000.

#### Goniopteris septemjuga (C.Chr.)

Taxon classificationPlantaePolypodialesThelypteridaceae

Salino & T.E.Almeida comb. nov.

urn:lsid:ipni.org:names:77151228-1

Dryopteris septemjuga C.Chr., Kongl. Svenska Vetenskapsakad. Handl., ser. 3, 16(2): 32, t. 7, f. 4-5. 1937.

#### Goniopteris skinneri (Hook.)

Taxon classificationPlantaePolypodialesThelypteridaceae

Salino & T.E.Almeida comb. nov.

urn:lsid:ipni.org:names:77151230-1

Aspidium skinneri Hook., Icon. Pl. 10: t. 924. 1854.
Thelypteris skinneri (Hook.) C.F.Reed, Phytologia 17(4): 314. 1968

#### Goniopteris stephanii (A.R.Sm. & M.Kessler)

Taxon classificationPlantaePolypodialesThelypteridaceae

Salino & T.E.Almeida comb. nov.

urn:lsid:ipni.org:names:77151232-1

Thelypteris stephanii A.R.Sm. & M.Kessler, Brittonia 60(1): 61. 2008.

#### Goniopteris stolzeana (A.R.Sm.)

Taxon classificationPlantaePolypodialesThelypteridaceae

Salino & T.E.Almeida comb. nov.

urn:lsid:ipni.org:names:77151333-1

Thelypteris stolzeana A.R.Sm., Phytologia 34(3): 231. 1976.

#### Goniopteris subsagittata (Maxon & C.Chr.)

Taxon classificationPlantaePolypodialesThelypteridaceae

Salino & T.E.Almeida comb. nov.

urn:lsid:ipni.org:names:77151253-1

Dryopteris subsagittata Maxon & C.Chr., Kongl. Svenska Vetenskapsakad. Handl., ser. 3, 16(2): 28, t. 6, f. 5–6. 1937.

#### Goniopteris tenebrica (Jenman)

Taxon classificationPlantaePolypodialesThelypteridaceae

Salino & T.E.Almeida comb. nov.

urn:lsid:ipni.org:names:77151234-1

Nephrodium tenebricum Jenman, J. Bot. 20: 326. 1882.
Thelypteris tenebrica (Jenman) Proctor, Bull. Inst. Jamaica, Sci. Ser. 5: 65. 1953.

#### Goniopteris tryoniorum (A.R.Sm.)

Taxon classificationPlantaePolypodialesThelypteridaceae

Salino & T.E.Almeida comb. nov.

urn:lsid:ipni.org:names:77151334-1

Thelypteris tryoniorum A.R.Sm., Fieldiana, Bot., n.s., 29: 62. 1992.

#### Goniopteris tuxtlensis (T.Krömer, Acebey & A.R.Sm.)

Taxon classificationPlantaePolypodialesThelypteridaceae

Salino & T.E.Almeida comb. nov.

urn:lsid:ipni.org:names:77151236-1

Thelypteris tuxtlensis T.Krömer, Acebey & A.R.Sm., Amer. Fern J. 97(3): 137–139. 2007.

#### Goniopteris urbani (Sodiro)

Taxon classificationPlantaePolypodialesThelypteridaceae

Salino & T.E.Almeida comb. nov.

urn:lsid:ipni.org:names:77151335-1

Polypodium urbani Sodiro, Crypt. Vasc. Quit. 301. 1893.
Thelypteris urbani (Sodiro) A.R.Sm., Fl. Ecuador 18: 116. 1983

#### Goniopteris verecunda (Proctor)

Taxon classificationPlantaePolypodialesThelypteridaceae

Salino & T.E.Almeida comb. nov.

urn:lsid:ipni.org:names:77151237-1

Thelypteris verecunda Proctor, Amer. Fern J. 75(2): 65. f 5. 1985.

#### Goniopteris yaucoensis (Proctor)

Taxon classificationPlantaePolypodialesThelypteridaceae

Salino & T.E.Almeida comb. nov.

urn:lsid:ipni.org:names:77151336-1

Thelypteris yaucoensis Proctor, Mem. New York Bot. Gard. 53: 208. 1989.

### New combinations in Steiropteris

#### Steiropteris subg. Glaphyropteris (Fée)

Taxon classificationPlantaePolypodialesThelypteridaceae

A.R.Sm. comb. et stat. nov.

urn:lsid:ipni.org:names:77151337-1

Glaphyropteris C.Presl ex Fée, Crypt. Vasc. Brésil, 2. 40. 1873.
Dryopteris subg. Glaphyropteris (C.Presl. ex Fée) C.Chr., Biol. Arb. Til. Eug. Warming 80. 1911.
Thelypteris subg. Glaphyropteris (C.Presl. ex Fée), Alston, J. Wash. Acad. Sci. 48: 234. 1958.

#### Steiropteris buchtienii (A.R.Sm.)

Taxon classificationPlantaePolypodialesThelypteridaceae

Salino & T.E.Almeida comb. nov.

urn:lsid:ipni.org:names:77151338-1

Thelypteris buchtienii A.R.Sm., Univ. Calif. Publ. Bot. 76: 10. 1980.

#### Steiropteris clypeolutata (Desv.) Pic.Serm. var. holmei (Baker)

Taxon classificationPlantaePolypodialesThelypteridaceae

Salino & T.E.Almeida comb. nov.

urn:lsid:ipni.org:names:77151240-1

Nephrodium holmei Baker, Ann. Bot. 5: 317. 1891.
Thelypteris clypeolutata (Desv.) Proctor var. holmei (Baker) A.R.Sm., Univ. Calif. Publ. Bot. 76: 11. 1980.

#### Steiropteris comosa (C.V.Morton)

Taxon classificationPlantaePolypodialesThelypteridaceae

Salino & T.E.Almeida comb. nov.

urn:lsid:ipni.org:names:77151242-1

Dryopteris comosa C.V.Morton, J. Wash. Acad. Sci. 28: 528. 1938.
Thelypteris comosa (C.V.Morton) C.V.Morton, Amer. Fern J. 51(1): 38. 1961.

#### Steiropteris decussata (L.)

Taxon classificationPlantaePolypodialesThelypteridaceae

A.R.Sm. comb. nov.

urn:lsid:ipni.org:names:77151339-1

Polypodium decussatum L., Species Plantarum 2: 1093. 1753.
Thelypteris decussata (L.) Proctor, Bull. Inst. Jamaica, Sci. Ser. 5: 59. 1953.

#### Steiropteris decussata (L.) A.R.Sm. var. brasiliensis (C.Chr.)

Taxon classificationPlantaePolypodialesThelypteridaceae

Salino & T.E.Almeida comb. nov.

urn:lsid:ipni.org:names:77151340-1

Dryopteris decussata var. brasiliensis C.Chr., Kongel. Danske Vidensk. Selsk. Skr., Naturvidensk. Math. Afd., ser. 7, 10: 161. 1913.
Thelypteris decussata var. brasiliensis (C.Chr.) A.R.Sm., Univ. Calif. Publ. Bot. 76: 15. 1980.

#### Steiropteris decussata var. costaricensis (A.R.Sm.)

Taxon classificationPlantaePolypodialesThelypteridaceae

Salino & T.E.Almeida comb. nov.

urn:lsid:ipni.org:names:77151341-1

Thelypteris decussata var. costaricensis A.R.Sm., Univ. Calif. Publ. Bot. 76: 16. 1980.

#### Steiropteris decussata var. mapiriensis (Rosenst.)

Taxon classificationPlantaePolypodialesThelypteridaceae

Salino & T.E.Almeida comb. nov.

urn:lsid:ipni.org:names:77151342-1

Dryopteris mapiriensis Rosenst., Repert. Spec. Nov. Regni Veg. 6: 313. 1909.
Thelypteris decussata var. mapiriensis (Rosenst.) A.R.Sm., Univ. Calif. Publ. Bot. 76: 16. 1980.

#### Steiropteris decussata var. velutina (Sodiro)

Taxon classificationPlantaePolypodialesThelypteridaceae

Salino & T.E.Almeida comb. nov.

urn:lsid:ipni.org:names:77151343-1

Polypodium velutinum Sodiro, Recens. Crypt. Vasc. Quit. 59. 1883.
Thelypteris decussata var. velutina (Sodiro) A.R.Sm., Univ. Calif. Publ. Bot. 76: 17. 1980.

#### Steiropteris fraseri (Mett. ex Kuhn)

Taxon classificationPlantaePolypodialesThelypteridaceae

Salino & T.E.Almeida comb. nov.

urn:lsid:ipni.org:names:77151344-1

Aspidium fraseri Mett. ex Kuhn, Linnaea 36: 109. 1869.
Thelypteris fraseri (Mett. ex Kuhn) A.R.Sm., Univ. Calif. Publ. Bot. 76: 32. 1980.

#### Steiropteris glandulosa (Desv.) Pic.Serm. var. brachyodus (Kunze)

Taxon classificationPlantaePolypodialesThelypteridaceae

Salino & T.E.Almeida comb. nov.

urn:lsid:ipni.org:names:77151256-1

Polypodium brachyodus Kunze, Linnaea 9: 48. 1834.
Thelypteris glandulosa var. brachyodus (Kunze) A.R.Sm., Phytologia 34: 233. 1976.

#### Steiropteris glandulosa (Desv.) Pic.Serm. var. longipilosa (A.R.Sm.)

Taxon classificationPlantaePolypodialesThelypteridaceae

Salino & T.E.Almeida comb. nov.

urn:lsid:ipni.org:names:77151345-1

Thelypteris glandulosa (Desv.) Proctor var. longipilosa A.R.Sm., Univ. Calif. Publ. Bot. 76: 22. 1980.

#### Steiropteris hatschbachii (A.R.Sm.)

Taxon classificationPlantaePolypodialesThelypteridaceae

Salino & T.E.Almeida comb. nov.

urn:lsid:ipni.org:names:77151346-1

Thelypteris hatschbachii A.R.Sm., Univ. Calif. Publ. Bot. 76: 22. 1980.

#### Steiropteris hottensis (C.Chr.)

Taxon classificationPlantaePolypodialesThelypteridaceae

Salino & T.E.Almeida comb. nov.

urn:lsid:ipni.org:names:77151244-1

Dryopteris hottensis C.Chr., Kongl. Svenska Vetenskapsacad. Handl., ser. 3, 16(2): 26. t 2(4-6). 1937.
Thelypteris hottensis (C.Chr.) A.R.Sm., Univ. Calif. Publ. Bot. 76: 23. 1980.

#### Steiropteris leprieurii (Hook.) Pic.Serm. var. costalis (Baker)

Taxon classificationPlantaePolypodialesThelypteridaceae

A.R.Sm. comb. nov.

urn:lsid:ipni.org:names:77151347-1

Nephrodium costale Baker, Syn Fil, 2. 495.1874.
Thelypteris leprieurii (Hook.) R.M.Tryon var. costalis (Baker) A.R.Sm., Univ. Calif. Publ. Bot. 76: 25. 1980.

#### Steiropteris leprieurii (Hook.) Pic.Serm. var. glandifera (A.R.Sm)

Taxon classificationPlantaePolypodialesThelypteridaceae

A.R.Sm. comb. nov.

urn:lsid:ipni.org:names:77151348-1

Thelypteris leprieurii (Hook.) R.M.Tryon var. glandifera A.R.Sm., Univ. Calif. Publ. Bot. 76: 25. 1980.

#### Steiropteris leprieurii (Hook.) Pic.Serm. var. incana (Christ)

Taxon classificationPlantaePolypodialesThelypteridaceae

A.R.Sm. comb. nov.

urn:lsid:ipni.org:names:77151258-1

Aspidium incanum Christ, Hedwigia 44: 367. 1905.
Thelypteris leprieurii (Hook.) R.M.Tryon var. incana (Christ) A.R.Sm., Univ. Calif. Publ. Bot. 76: 26. 1980.

#### Steiropteris leprieurii (Hook.) Pic.Serm. var. subcostalis (A.R.Sm.)

Taxon classificationPlantaePolypodialesThelypteridaceae

A.R.Sm. comb. nov.

urn:lsid:ipni.org:names:77151349-1

Thelypteris leprieurii (Hook.) R.M.Tryon var. subcostalis A.R.Sm., Univ. Calif. Publ. Bot. 76: 26. 1980.

#### Steiropteris mexiae (C.Chr. ex Copel.)

Taxon classificationPlantaePolypodialesThelypteridaceae

Salino & T.E.Almeida comb. nov.

urn:lsid:ipni.org:names:77151246-1

Dryopteris mexiae C.Chr. ex Copel., Univ. Calif. Publ. Bot., 17: 32. 1932.
Thelypteris mexiae (C.Chr. ex Copel.) Ching, Bull. Fan Mem. Inst. Biol. 10: 252. 1941.

#### Steiropteris parva (A.R.Sm. & M.Kessler)

Taxon classificationPlantaePolypodialesThelypteridaceae

Salino & T.E.Almeida comb. nov.

urn:lsid:ipni.org:names:77151350-1

Thelypteris parva A.R.Sm. & M.Kessler, Brittonia 60(1): 57. 2008.

#### Steiropteris pennellii (A.R.Sm.)

Taxon classificationPlantaePolypodialesThelypteridaceae

Salino & T.E.Almeida comb. nov.

urn:lsid:ipni.org:names:77151351-1

Thelypteris pennellii A.R.Sm., Univ. Calif. Publ. Bot. 76: 28. 1980.

#### Steiropteris polyphlebia (C.Chr.)

Taxon classificationPlantaePolypodialesThelypteridaceae

Salino & T.E.Almeida comb. nov.

urn:lsid:ipni.org:names:77151248-1

Dryopteris polyphlebia C.Chr., Kongel. Danske Vidensk. Selsk. Skr., Naturvidensk. Math. Afd., ser. 7, 10: 161. 1913.
Thelypteris polyphlebia (C.Chr.) C.V.Morton, Amer. Fern J. 51(1): 38. 1961.

#### Steiropteris polypodioides (Raddi)

Taxon classificationPlantaePolypodialesThelypteridaceae

Salino & T.E.Almeida comb. nov.

urn:lsid:ipni.org:names:77151250-1

Ceterach polypodioides Raddi, Opusc. Sci. 3: 284. 1819.
Thelypteris polypodioides (Raddi) C.F.Reed, Phytologia 7: 305. 1968.

#### Steiropteris seemannii (J.Sm.)

Taxon classificationPlantaePolypodialesThelypteridaceae

Salino & T.E.Almeida comb. nov.

urn:lsid:ipni.org:names:77151353-1

Phegopteris seemannii J.Sm., in Seem., Bot. Voy. Herald. 228. 1854.
Thelypteris seemannii (J.Sm.) A.R.Sm., Univ. Calif. Publ. Bot. 76: 30. 1980.

#### Steiropteris villosa (Link)

Taxon classificationPlantaePolypodialesThelypteridaceae

Salino & T.E.Almeida comb. nov.

urn:lsid:ipni.org:names:77151252-1

Gymnogramma villosa Link, Hort. Berol. 2: 51. 1833.
Thelypteris villosa (Link) C.F.Reed, Phytologia 7: 323. 1968.
